# Carrier-free nanoparticles of camptothecin prodrug for chemo-photothermal therapy: the making, in vitro and in vivo testing

**DOI:** 10.1186/s12951-021-01093-y

**Published:** 2021-10-30

**Authors:** Mingtao Ao, Fei Yu, Yixiang Li, Mengya Zhong, Yonghe Tang, Hua Yang, Xiaojing Wu, Yifan Zhuang, Huiyun Wang, Xiaolian Sun, Xuehui Hong, Xiao Dong Chen

**Affiliations:** 1 Medical College, Guangxi University, Nanning, 530004 China; 2 Suzhou Key Lab of Green Chemical Engineering, School of Chemical and Environmental Engineering, College of Chemistry, Chemical Engineering and Materials Science, Soochow University, Suzhou, 215123 China; 3 Department of Gastrointestinal Surgery, Zhongshan Hospital of Xiamen University, Xiamen, 361005 China; 4 Department of Pharmacy, Jining Medical University, Rizhao, 276826 China; 5 State Key Laboratory of Natural Medicines, Key Laboratory of Drug Quality Control and Pharmacovigilance, Department of Pharmaceutical Analysis, China Pharmaceutical University, Nanjing, 210009 China; 6 Guangxi Key Laboratory of Electrochemical Energy Materials, Guangxi University, Nanning, 530004 China; 7 School of Pharmaceutical Sciences, Xiamen University, Xiamen, 361102 China

**Keywords:** Camptothecin, IR820, Nanoparticles, Prodrug, Controlled release

## Abstract

**Background:**

Nanoscale drug delivery systems have emerged as broadly applicable approach for chemo-photothermal therapy. However, these nanoscale drug delivery systems suffer from carrier-induced toxicity, uncontrolled drug release and low drug carrying capacity issues. Thus, to develop carrier-free nanoparticles self-assembled from amphiphilic drug molecules, containing photothermal agent and anticancer drug, are very attractive.

**Results:**

In this study, we conjugated camptothecin (CPT) with a photothermal agent new indocyanine green (IR820) via a redox-responsive disulfide linker. The resulting amphiphilic drug–drug conjugate (IR820-SS-CPT) can self-assemble into nanoparticles (IR820-SS-CPT NPs) in aqueous solution, thus remarkably improving the membrane permeability of IR820 and the aqueous solubility of CPT. The disulfide bond in the IR820-SS-CPT NPs could be cleaved in GSH rich tumor microenvironment, leading to the on demand release of the conjugated drug. Importantly, the IR820-SS-CPT NPs displayed an extremely high therapeutic agent loading efficiency (approaching 100%). Besides, in vitro experimental results indicated that IR820-SS-CPT NPs displayed remarkable tumor cell killing efficiency. Especially, the IR820-SS-CPT NPs exhibited excellent anti-tumor effects in vivo. Both in vitro and in vivo experiments were conducted, which have indicated that the design of IR820-SS-CPT NPs can provide an efficient nanotherapeutics for chemo-photothermal therapy.

**Conclusion:**

A novel activatable amphiphilic small molecular prodrug IR820-SS-CPT has been developed in this study, which integrated multiple advantages of GSH-triggered drug release, high therapeutic agent content, and combined chemo-photothermal therapy into one drug delivery system.

**Graphical Abstract:**

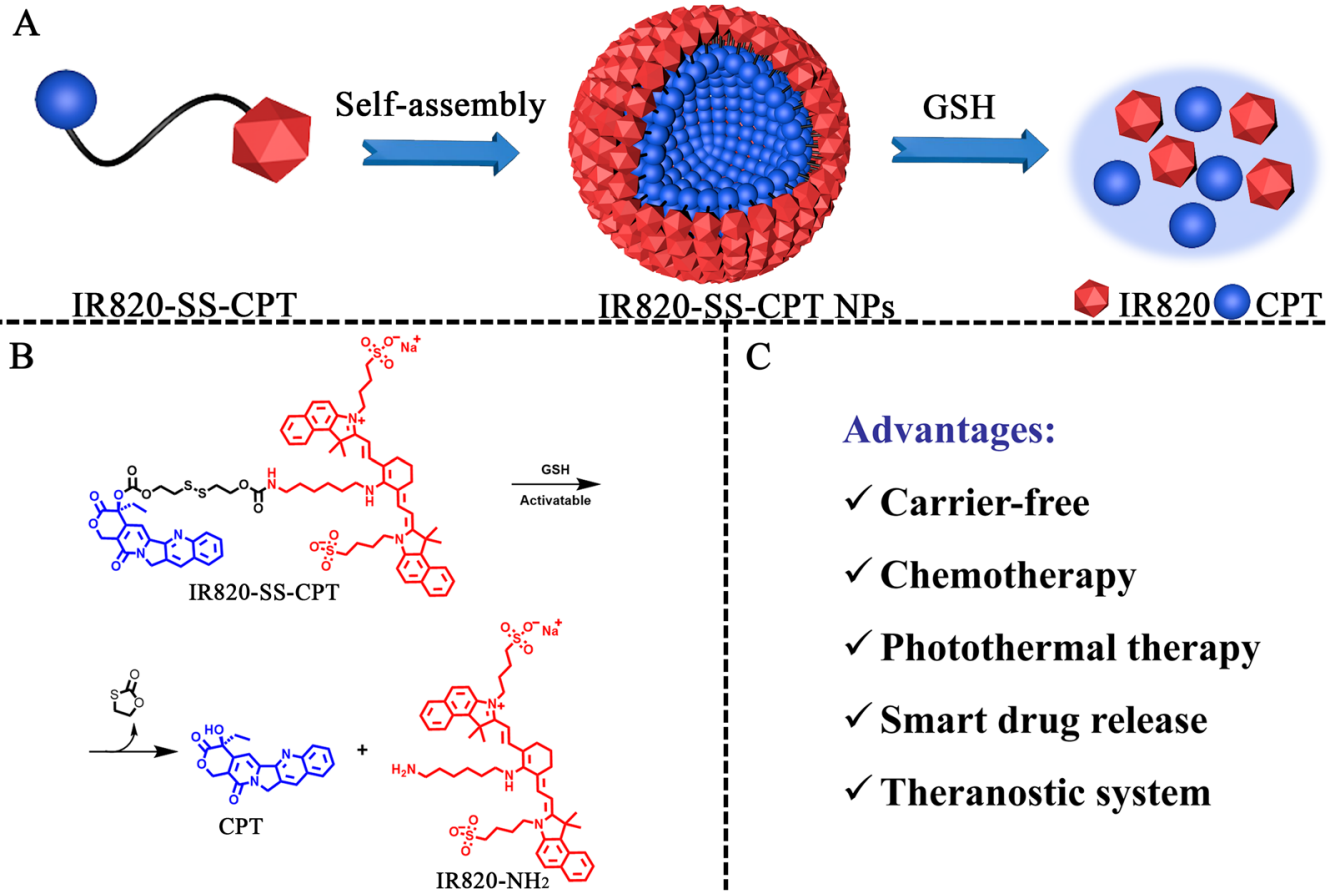

**Supplementary Information:**

The online version contains supplementary material available at 10.1186/s12951-021-01093-y.

## Background

The incidence of cancers, as one of leading cause of death, is a severe public health problem [1, 2]. Until now, chemotherapy is the major treatment strategy for cancers [3, 4]. However, chemotherapy itself has many disadvantages in clinical practice, such as poor therapeutic efficacy, systemic toxicity, and drug resistance [5, 6]. To address these issues, there has been a great interest in building synergistic drug delivery systems capable of co-delivery of two or more therapeutic agents to achieve synergistic therapeutic effect for battling the tumor heterogeneity [7, 8]. These strategies may not only take advantage of different treatments to remarkably augment the anti-tumor effect, but can also overcome serious adverse effects [9, 10]. Recently, the combination of chemotherapy and photothermal therapy has attracted great attention in the field of cancer treatment [11, 12]. Photothermal therapy (PTT), using near-infrared (NIR) photothermal light agents to absorb light energy, could lead to irreversible tumor injury [13, 14]. As a promising invasive treatment strategy for cancers [15, 16], the hyperthermia could also improve tumor cellular uptake of antitumor drugs via increasing the permeability of cell membrane [17, 18], and improve the sensitivity of cells towards chemotherapy [19, 20]. To achieve this, many nanoscale drug delivery systems have been constructed for the co-delivery of photothermal agents and chemotherapeutic agents for synergistic cancer therapy [21].

Although synergistic effects of these drug delivery systems have been proved by many studies, they have to be confronted with many limitations to wider applications. The current state of these drug delivery systems is greatly restricted by the excessive use of additional excipients, the carrier-related toxicity, and premature drug release during blood circulation [22–24]. Therefore, the development of new nanoparticles with high drug loading capacity, precise drug release at tumor sites and easy fabrication is highly desired.

Amphiphilic small molecular prodrugs, possessing apparent advantages of convenient assembly and high drug loading capacity, have been attracting great attention [25, 26]. Unlike the traditional nanoscale drug delivery systems, these amphiphilic small molecular prodrugs can self-assemble into nanoparticles without using any additional carriers, which could reduce the risk of potential long term toxicity [27, 28]. Yan et al. reported carrier-free Ir–Cb nanoparticles constructed with an amphiphilic drug–drug conjugate (ADDC) [29]. After uptake by the tumor cells, the carrier-free Ir–Cb nanoparticles could release free Ir and Cb with improved cancer therapeutic efficacy. Although the amphiphilic small molecular prodrugs are intriguing, they still exist many shortcomings. Most of these amphiphilic drug–drug conjugates were constructed through ester linkage, which could also be cleaved in the extracellular tumor tissues before cellular internalization, causing the decrease of anticancer efficacy. Besides, the ester bond is a rather stable linkage, which will lead to slow drug release in the intracellular environment and the reduction of cytotoxicity potency [30].

**Scheme 1:**
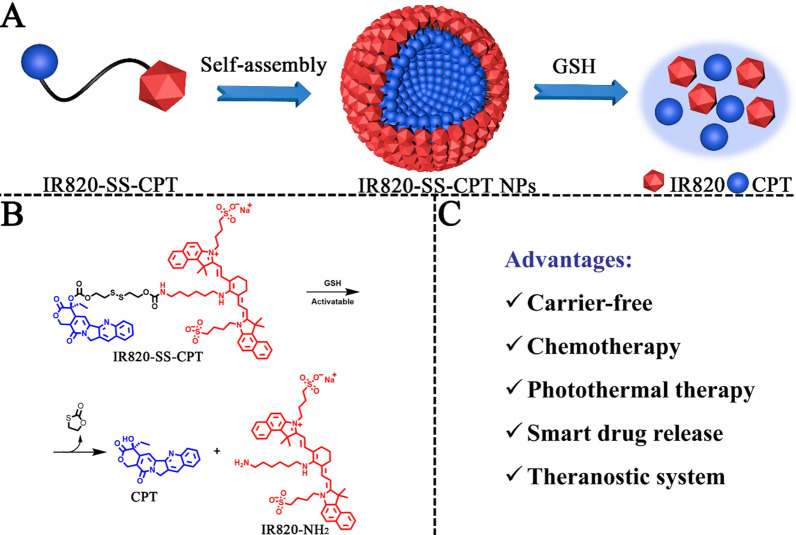
Illustration of the redox-responsive IR820-SS-CPT NPs for chemo-photothermal therapy. **A** Schematic diagram of self-assembly of the IR820-SS-CPT NPs via hydrophobic and π–π stacking interactions. **B** The speculated mechanisms of CPT release from the IR820-SS-CPT NPs by treatment of GSH. **C** Advantages of the proposed IR820-SS-CPT NPs

In this work, we used the redox-responsive disulfide bond to link the hydrophobic drug CPT with hydrophilic photothermal agent IR820 for forming a new kind of prodrug amphiphile, named as IR820-SS-CPT (Scheme 1). The constructed IR820-SS-CPT has been featured with several outstanding advantages. Firstly, owing to its amphiphilicity, the IR820-SS-CPT could self-assemble into nanoparticles (designated as IR820-SS-CPT NPs) in aqueous solution without the help of any other carriers. Secondly, the IR820-SS-CPT can remain stable under low concentration of glutathione (GSH) in blood circulation, so the side effects can be reduced. In addition, the disulfide bonds in IR820-SS-CPT can be rapidly cleaved by the elevated intracellular concentration of GSH in tumors, whereby transforming the IR820-SS-CPT NPs into highly toxic CPT. All these results suggest that the developed redox-sensitive IR820-SS-CPT NPs in this work are promising for cancer theranostics.

## Results and discussion

### Synthesis and characterization of IR820-SS-CPT conjugate

**Scheme 2:**
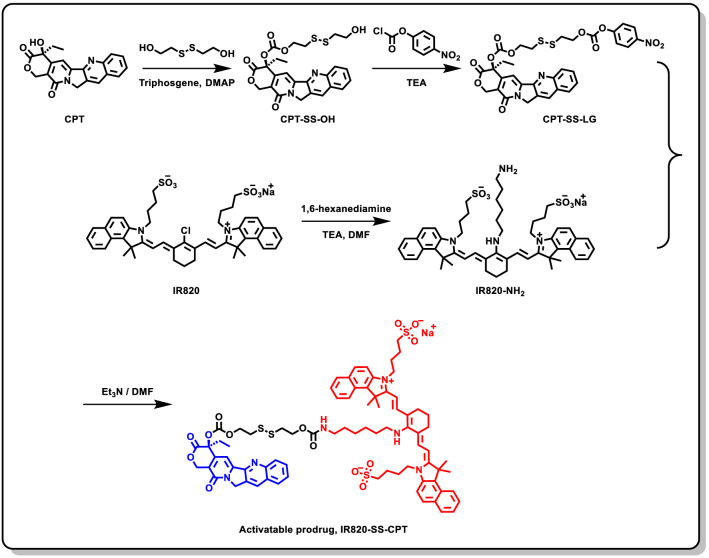
Synthesis routes of IR820-SS-CPT

As depicted in Scheme 2, the theranostic prodrug IR820-SS-CPT was synthesized through a four-step process. Firstly, CPT-SS-OH was prepared by the reaction of CPT with 2-hydroxyethyl disulfide in the presence of triphosgene [31, 32]. Next, the reaction between CPT-SS-OH and 4-nitrophenyl chloroformate gave the hydroxyl-activated ester CPT-SS-LG. Then, the key intermediate IR820-NH_2_ was obtained by the reaction of IR820 with 1,6-hexanediamine [33]. Finally, IR820-NH_2_ reacted with CPT-SS-LG to give IR820-SS-CPT. The chemical structure of IR820-SS-CPT was characterized by ^1^H NMR, ^13^C NMR, and ESI–MS. Additional file 1: Fig. S7 shows the ^1^H NMR spectra of the product IR820-SS-CPT. The characteristic peaks of ethyl group on the lactone ring of CPT were found at 2.16 (td, *J* = 7.2, 14.3 Hz, 2H) and 0.91 (t, *J* = 7.3 Hz, 3H) ppm. Meanwhile, the characteristic peaks of four methyl groups of IR820 appeared at 1.92–1.85 (m, 12H) ppm. Furthermore, the aromatic protons appeared at 7.0–9.0 ppm corresponding to the characteristic peaks of CPT and IR820 were also observed in Additional file 1: Fig. S7, indicating the successful synthesis of IR820-SS-CPT. Furthermore, ESI–MS was used to confirm the molecular weight of IR820-SS-CPT (Additional file 1: Fig. S9). The peaks (m/z = 1483.6 and 1461.4) were assigned as [M+H]^+^ and [M−Na+H]^+^, respectively. The UV–vis spectrum of the IR820-SS-CPT is displayed in Additional file 1: Fig. S14. Compared to the characteristic absorption peak of free IR820 at 821 nm, there was about a 160 nm blue-shift in the spectrum of IR820-SS-CPT at 660 nm. All the synthesis details and the corresponding characterization can be found in the Supporting Information, including the non-cleavable IR820-CC-CPT (Additional file 1: Figs. S1–S14).

### Preparation and characterization of IR820-SS-CPT NPs

**Fig. 1:**
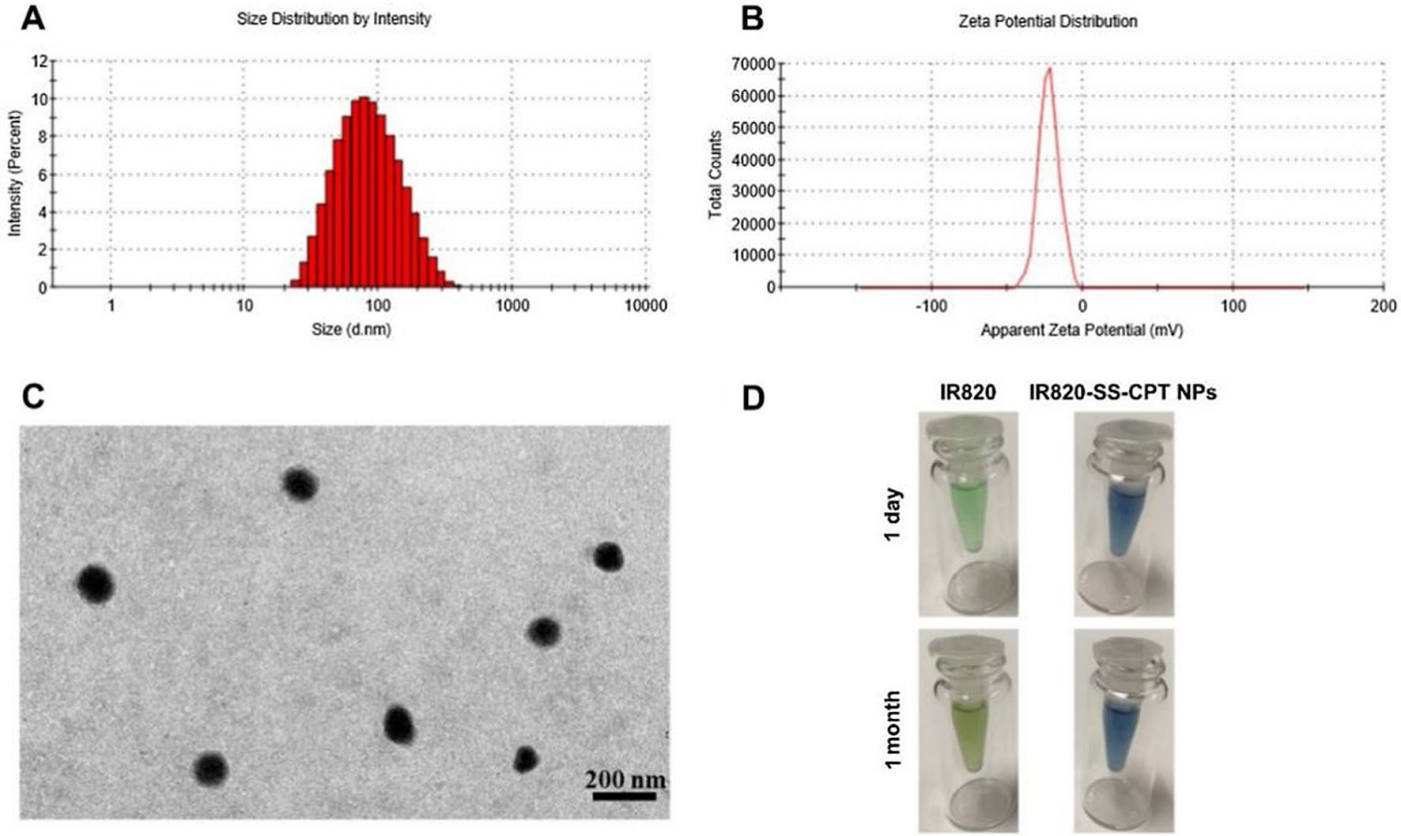
**A** Size distribution of IR820-SS-CPT NPs. **B** Zeta potential of IR820-SS-CPT NPs. **C** TEM image of IR820-SS-CPT NPs. **D** Photographs of free IR820 and IR820-SS-CPT NPs after being stored at room temperature

As it was shown in Fig. 1, the IR820-SS-CPT prodrug could self-assemble nanoparticles in water via self-assembly method due to its inherent amphiphilic structure. Besides, some weak intermolecular interactions in the IR820-SS-CPT conjugates such as π–π stacking interaction and hydrogen bond interaction could enhance the formation of IR820-SS-CPT NPs. The dynamic light scattering (DLS) and transmission electron microscopy (TEM) analyses were used to investigate the size distribution and morphology of IR820-SS-CPT NPs. The DLS data (Fig. 1A) indicated that the IR820-SS-CPT NPs possessed an average hydrodynamic diameter of approximate 72.5 nm with a narrow distribution (PDI = 0.248). In addition, IR820-SS-CPT NPs displayed a negatively charged (− 22.5 mV) surface in water solution (Fig. 1B). The TEM image (Fig. 1C) further demonstrated that the morphology of the IR820-SS-CPT NPs was spherical nanoparticles.

The stability of nanoparticles is also an important parameter. After 1-month storage at room temperature, there was few appearance change for the IR820-SS-CPT NPs water solution. However, obvious color change (from green to gray) was observed in the IR820 water solution (Fig. 1D). Apparently, the IR820-SS-CPT NPs displayed superior stability in aqueous solution than that of free IR820. The improved stability might be attributed to the effective encapsulation by IR820-SS-CPT NPs, which could protect it from the light. Besides, the nanoparticles could prevent the aggregation of IR820, which avoided the formation of dimers and oligomers. In addition, there were no obvious changes of the hydrodynamic diameter of IR820-SS-CPT NPs during the storage time (Additional file 1: Fig. S15). Taken together, the IR820-SS-CPT NPs could provide an excellent alternative option in biomedical applications.

### In vitro drug release and photothermal effects

**Fig. 2:**
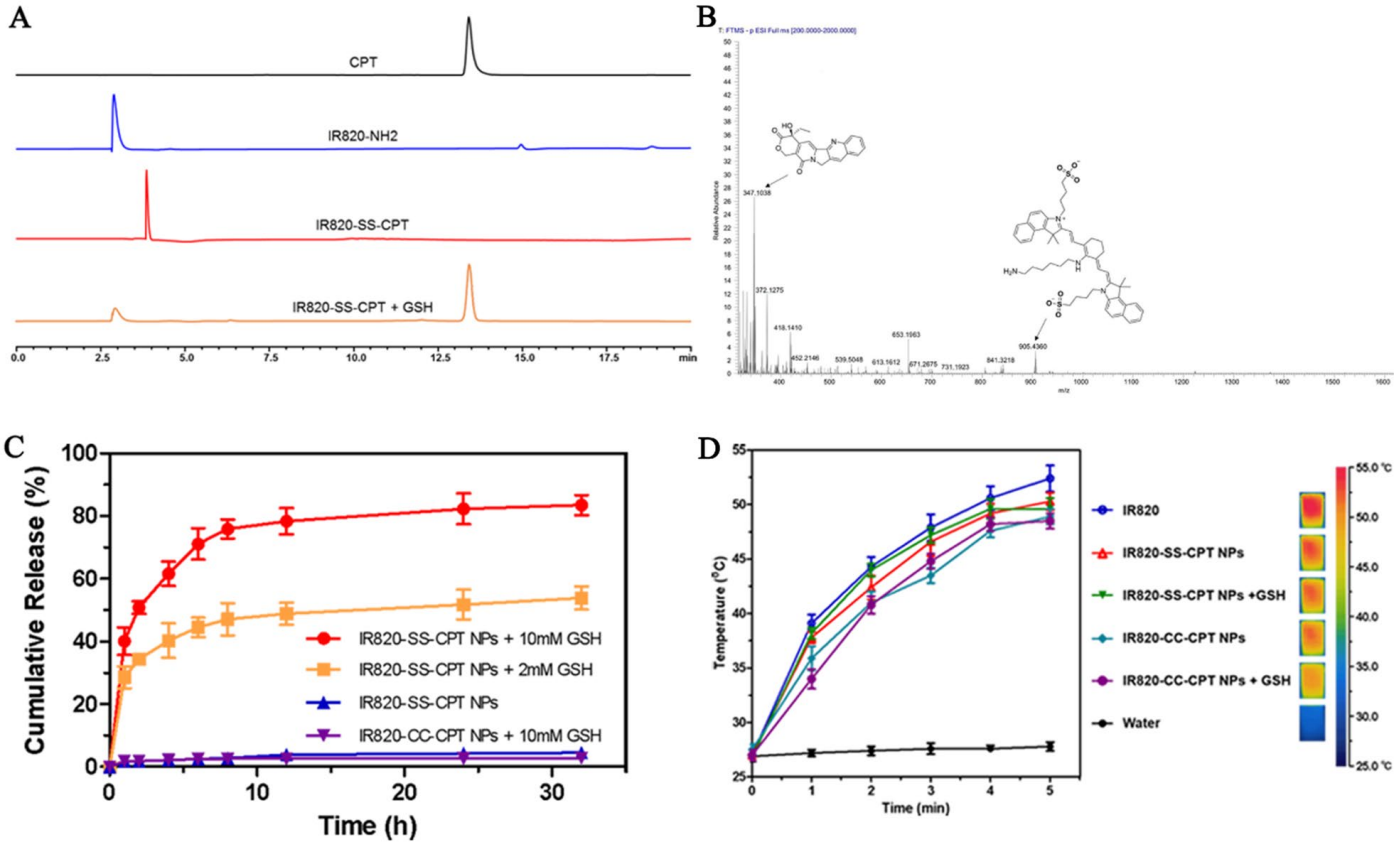
**A** HPLC chromatogram of the IR820-SS-CPT NPs in the presence of GSH (10 mM). **B** HRMS spectrum of the products from the reaction of IR820-SS-CPT with GSH (10 mM) in DMSO/H_2_O (1:99, v/v). **C** In vitro CPT release from the IR820-SS-CPT or IR820-CC-CPT NPs in the presence of various amount of GSH. **D** Temperature increase profiles of IR820, IR820-SS-CPT NPs, IR820-SS-CPT NPs + GSH, IR820-CC-CPT NPs, and IR820-CC-CPT NPs + GSH with laser irradiation for 5 min (IR820-SS-CPT NPs: 660 nm, 1.0 W/cm^2^; IR820: 808 nm, 1.0 W/cm^2^). The equivalent IR820 dose was kept at 100 µM

HPLC analyses were applied to investigate the anticipated release behavior of CPT from IR820-SS-CPT NPs. As shown in Fig. 2A, after the IR820-SS-CPT NPs were incubated with GSH, the peak of free CPT was observed in HPLC chromatogram. This indicated that the active cancer drug release could be achieved for the IR820-SS-CPT NPs. This finding was also confirmed by the high resolution MS analyses (Fig. 2B). The ionic peak of 347.1038 (corresponding to [CPT–H]^−^) was also observed in the high resolution MS spectrum.

To investigate the effects of different GSH concentrations on the CPT release from the IR820-SS-CPT NPs, in vitro cumulative drug release profiles were conducted in different media at 37 °C. As shown in Fig. 2C, about 83.5% of CPT was released from the IR820-SS-CPT NPs in the presence of 10 mM GSH (equivalent to a tumoral environment) after incubation for 32 h, while the release of CPT was approximately 53.9% in the presence of 2 mM GSH. In comparison, less than 4% of CPT release was obtained at 32 h without GSH, exhibiting a very low release rate. These results indicated IR820-SS-CPT NPs could realize the redox-responsive release behavior of CPT in tumoral environment. Similarly, IR820-SS-CPT NPs could decrease the unfavorable drug release during blood circulation and increase the desired selective drug release in tumor. Apparently, this IR820-SS-CPT NPs exhibited its great potential for loading CPT in a stimuli-responsive way.

As shown in Additional file 1: Fig. S16, very weak inherent fluorescence of CPT in the IR820-SS-CPT NPs was observed, which was attributed to the fluorescence was quenched by the self-assembled nanoparticles. In contrast, the fluorescence emission intensity of IR820-SS-CPT NPs at 430 nm increased drastically after the addition of GSH. The inherent fluorescence recovery of CPT was ascribed to the destroyed disulfide bond under high GSH concentration and subsequent disassembly of IR820-SS-CPT NPs. Apparently, the IR820-SS-CPT NPs could monitor the CPT drug release as a fluorescence switch.

To investigate the photothermal efficiency of the IR820-SS-CPT NPs in PTT, the temperature changes were recorded in vitro under laser irradiation at 660 nm. Upon being irradiated with 1.0 W/cm^2^ for 5 min, the temperature of IR820-SS-CPT NPs and IR820 rapidly increased to 50.3 °C and 52.4 °C respectively, while the water displayed only a slight temperature increase (Fig. 2D). Such temperature increase of IR820-SS-CPT NPs (over 43 °C) will lead to an irreversible damage to tumor cells. In addition, the temperature of the IR820-SS-CPT NPs increased slower than that of free IR820 aqueous solution at the same concentration under the irradiation. These results might be attributed to the formation of the IR820-SS-CPT NPs, which weakened the absorption of IR820 in NIR region. This was consistent with the results of the infrared thermal images. Meanwhile, after being interacted with GSH, there was no much difference for the temperature elevation.

### In vitro cellular uptake

**Fig. 3:**
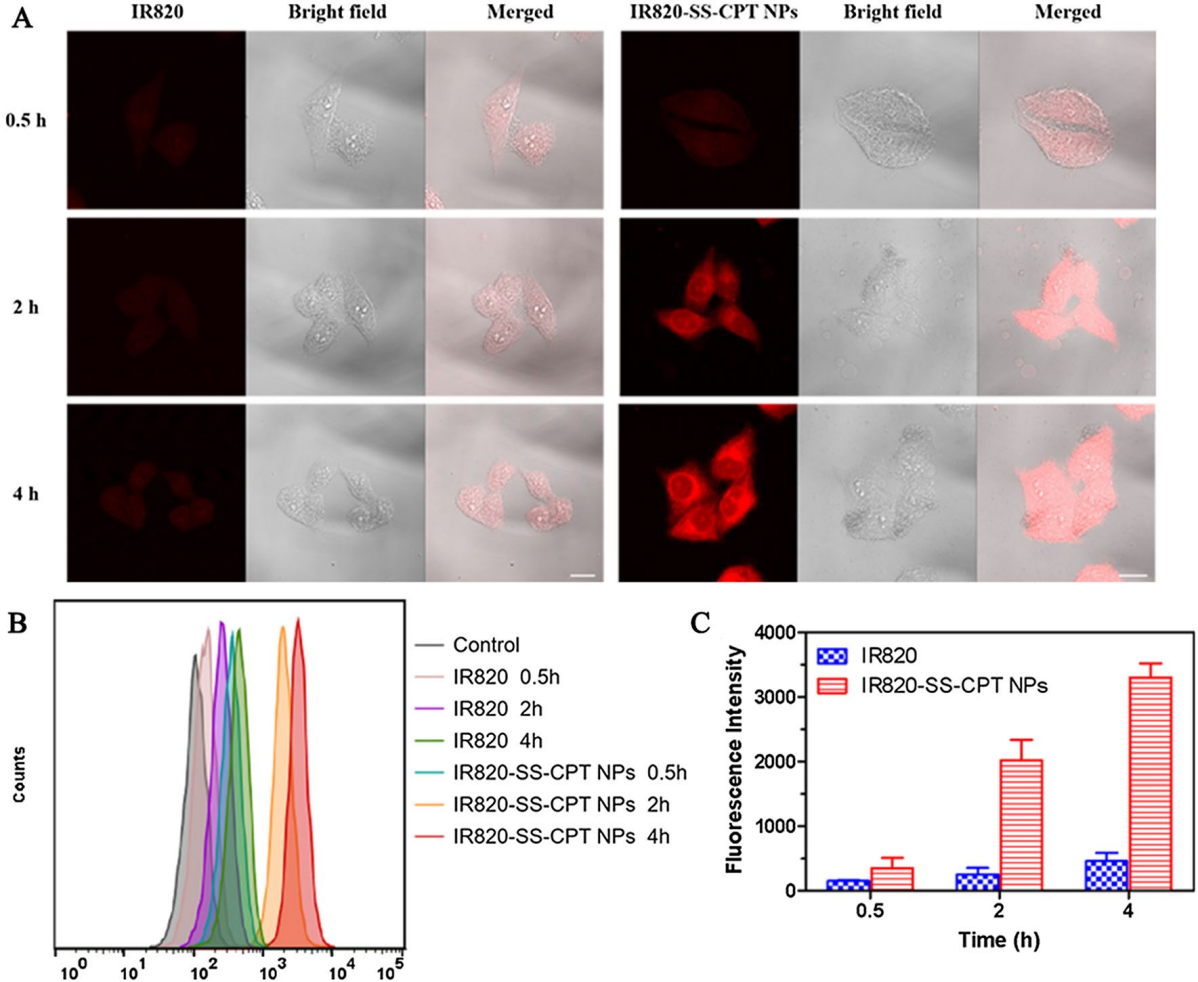
In vitro cellular uptake of IR820-SS-CPT NPs. **A** LSCM images of 4T1 cells incubated with IR820 or IR820-SS-CPT NPs for 0.5, 2, and 4 h. Red represents the fluorescence of IR820 (scale bar = 20 µm). The wavelength of excitation laser was set at 633 nm. **B** Flow cytometry analysis of 4T1 cells incubated with free IR820 and IR820-SS-CPT NPs for 0.5, 2, and 4 h. **C** Mean fluorescent intensity of free IR820 and IR820-SS-CPT NPs internalized by 4T1 cells after incubation for 0.5, 2, and 4 h

To investigate the effective cellular uptake of IR820-SS-CPT NPs, the internalized behavior of the IR820-SS-CPT NPs in 4T1 cancer cells was evaluated by flow cytometry and laser scanning confocal microscopy (LSCM). IR820 was used as a fluorescent indicator. As shown in Fig. 3A, after being interacted with IR820-SS-CPT NPs, the fluorescence intensity of the 4T1 cancer cells gradually increased over time. Besides, the IR820-SS-CPT NPs group displayed stronger fluorescent intensity than that of free IR820 group. This was attributed to the IR820-SS-CPT NPs could enter the 4T1 cells based on the endocytosis mechanism, which was more effective than that of passive diffusion. This result was also consistent with the quantitative analysis by flow cytometry (Fig. 3B, C).

Chlorpromazine, amiloride, and nystatin were used to investigate the in vitro cell uptake mechanism. As shown in Additional file 1: Fig. S17, both nystatin and chlorpromazine induced the decrease of cellular uptake of IR820-SS-CPT NPs, suggesting that the caveolae- and clathrin-mediated endocytotic pathway mediated the internalization of the IR820-SS-CPT NPs into 4T1 cells. Meanwhile, when incubated at 4 °C, the uptake of IR820-SS-CPT NPs decreased significantly, implying that the processes of the endocytotic cellular uptake were ATP- and energy-dependent.

### In vitro antitumor study

**Fig. 4:**
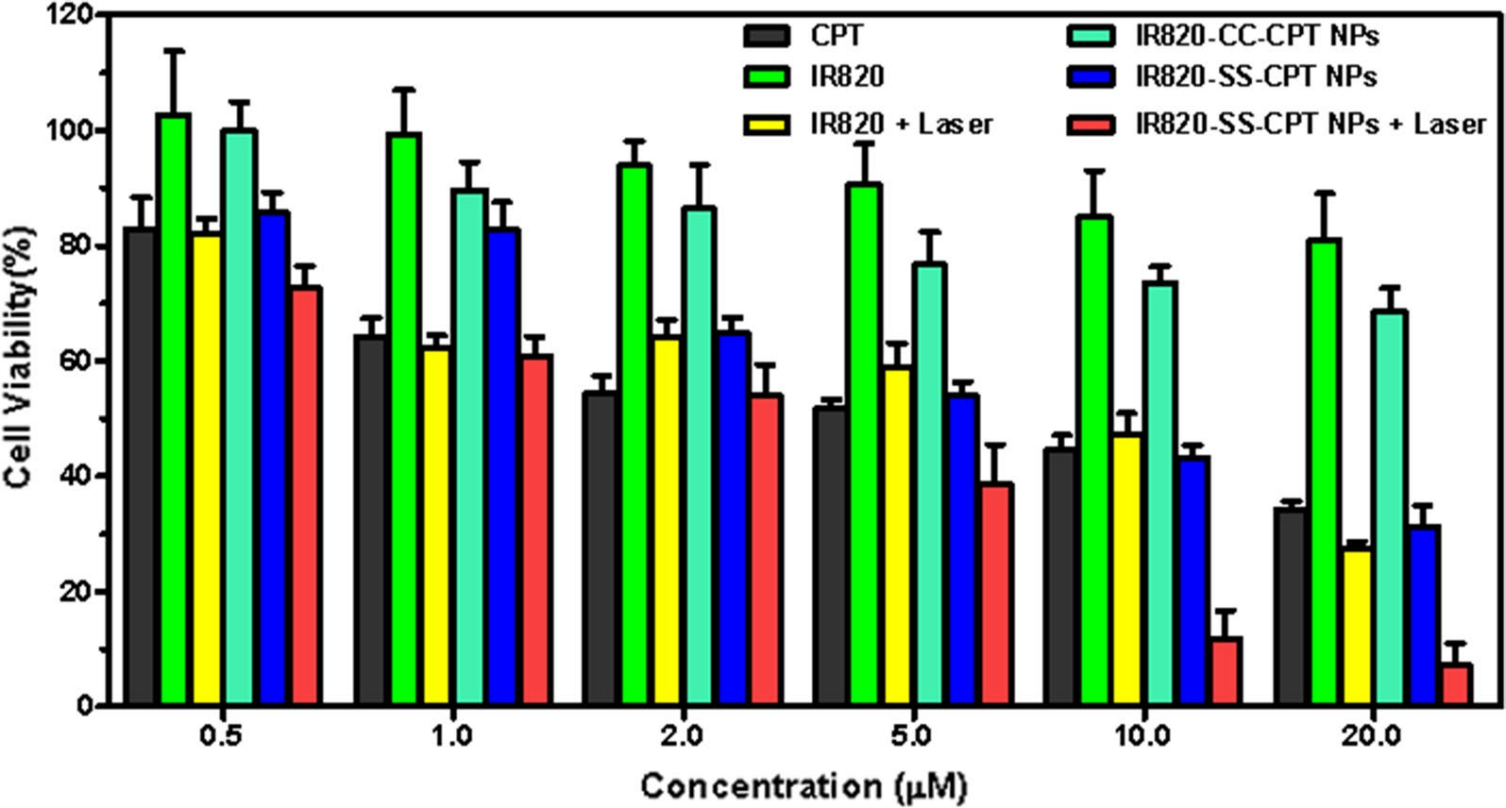
In vitro cell survivals. Relative viability of 4T1 cells treated with various concentrations of CPT, free IR820, free IR820 + Laser, IR820-CC-CPT NPs, IR820-SS-CPT NPs, or IR820-SS-CPT NPs + Laser for 36 h (IR820-SS-CPT NPs: 660 nm, 1.0 W/cm^2^; IR820: 808 nm, 1.0 W/cm^2^; t = 5 min)

To evaluate the chemo-photothermal therapeutic efficiency of the IR820-SS-CPT NPs in vitro, 4T1 cells were treated with or without irradiation (5 min, 1.0 W/cm^2^) in the presence of IR820-SS-CPT NPs. The cell viability of 4T1 cells was measured using MTT assay. As shown in Fig. 4, all the groups displayed a dose-dependent inhibition effect manner. In addition, it should be noted that the IR820-SS-CPT NPs + laser group displayed the highest cytotoxicity (IC_50_, 2.39 ± 0.92 μM). This result indicated that the IR820-SS-CPT NPs displayed superior inhibition to the 4T1 cells with laser irradiation than others.

Besides, to further investigate the therapeutic selectivity of IR820-SS-CPT NPs, the in vitro cytotoxicity was also evaluated by the LO2 cells. As shown in Additional file 1: Fig. S18, free CPT still displayed identical cytotoxicity against LO2 cells, which would induce the potential side effects. In contrast, the IR820-SS-CPT NPs displayed lower cytotoxicity to LO2 cells, which was attributed to the low concentrations of GSH in normal cells. Based on the excellent performances of in vitro cytotoxicity and redox-responsive release behavior, the IR820-SS-CPT NPs would be investigated in the following study.

### In vivo imaging and biodistribution

**Fig. 5:**
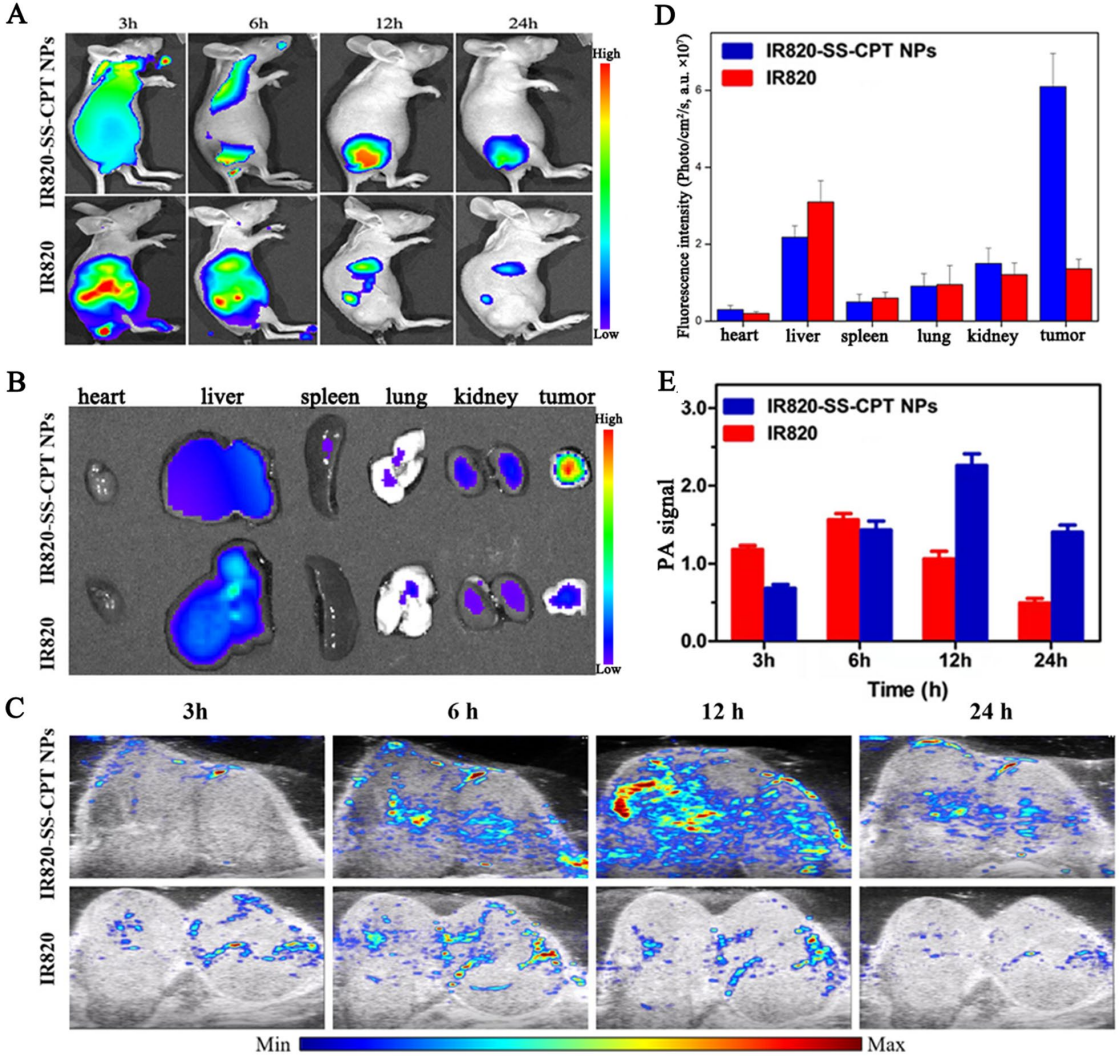
In vivo biodistribution and molecular imaging of IR820-SS-CPT NPs and free IR820. **A** In vivo NIR fluorescence images of 4T1 tumor-bearing nude mice by intravenous administration of IR820-SS-CPT NPs or free IR820 for 3, 6, 12, and 24 h. **B** Ex vivo fluorescence images of tumors and major organs at 24 h post-injection of IR820-SS-CPT NPs or free IR820. **C** PA images of the mice injected with IR820-SS-CPT NPs or free IR820 for 3, 6, 12, and 24 h. **D** Fluorescence intensity of tumors and major organs. **E** Photoacoustic signals in the tumors of the mice at different time points

The NIR fluorescence imaging in vivo was used as an ideal tool to investigate the passive tumor-targeting capability of IR820-SS-CPT NPs (Fig. 5A). 4T1 tumor-bearing BALB/c nude mice were intravenously injected with IR820-SS-CPT NPs. At predetermined time intervals, the in vivo images were obtained to record the in vivo biodistribution of IR820-SS-CPT NPs. As displayed in Fig. 5A, in the IR820-SS-CPT NPs group, the fluorescence signal could be observed at 3 h. Subsequently, the strongest fluorescence signals of IR820-SS-CPT NPs could be observed at 12 h. On the contrary, a much weaker fluorescence signal was observed at the tumor site in the free IR820 group. This result implied that the IR820-SS-CPT NPs could passively accumulate in the tumor efficiently via EPR effect. Besides, the ex vivo finding was in accordance with the results of the in vivo study (Fig. 5B, D). As the important metabolic organ, the fluorescence intensity of free IR820 in liver was very high compared with other organs. In addition, owing to the EPR effect, the IR820-SS-CPT NPs displayed the higher fluorescence intensity in tumors.

Meanwhile, the photoacoustic images of 4T1 tumor-bearing nude mice after i.v. injection of IR820-SS-CPT NPs were also recorded at different times for visualizing the accumulation behavior. As shown in Fig. 5C, E, photoacoustic signals of IR820-SS-CPT NPs were stronger than that of free IR820, reaching a maximum at 12 h. This result further demonstrated the efficient tumor homing of IR820-SS-CPT NPs, which was in accordance with the observation of the NIR fluorescence imaging in vivo.

### In vivo pharmacokinetics

Usually, the nanoparticles (< 200 nm), unlike free small molecular drugs, would possess excellent retention time in the bloodstream. The pharmacokinetic study was conducted to confirm this hypothesis. The blood samples of the Sprague–Dawley (SD) rats (∼ 200 g) were determined at predetermined time points. As shown in Additional file 1: Fig. S19, the concentration of IR820-SS-CPT NPs (with a half-life of ∼ 1.75 h) decreased slower over time compared with that of CPT (with a half-life of ∼ 0.19 h). This result indicated that the IR820-SS-CPT NPs possessed longer blood retention time, which would be beneficial to the accumulation of IR820-SS-CPT NPs at tumor tissues.

### In vivo combinational therapy of IR820-SS-CPT NPs

**Fig. 6:**
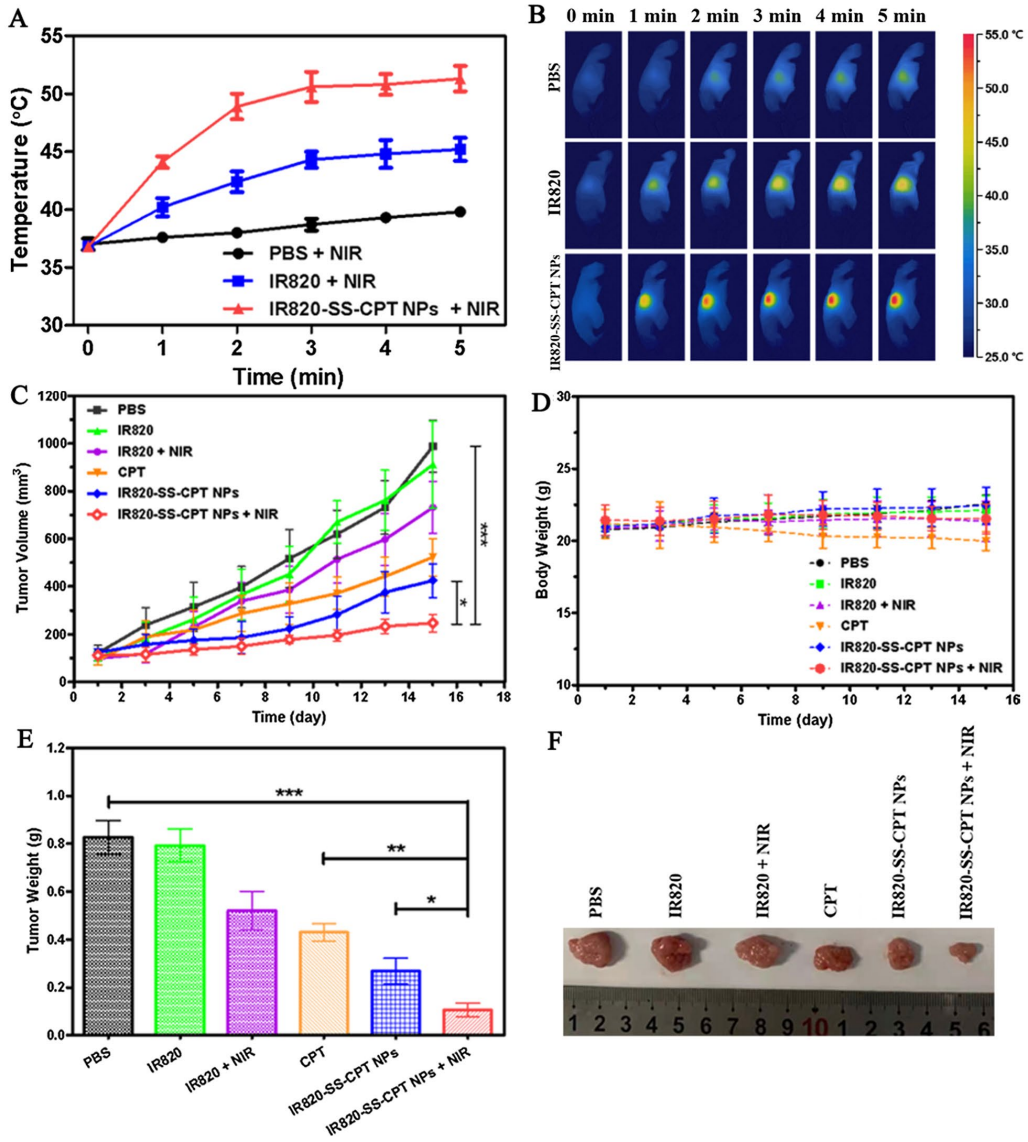
In vivo antitumor performance and safety evaluation in the 4T1 tumor-bearing mice. Temperature increase profiles (**A**) and infrared thermal images (**B**) of the 4T1 tumor-bearing mice irradiated with laser (IR820-SS-CPT NPs: 660 nm, 1.0 W/cm^2^; IR820: 808 nm, 1.0 W/cm^2^). **C** Tumor volume changes of the mice treated with PBS, IR820, IR820 + NIR, CPT, IR820-SS-CPT NPs, and IR820-SS-CPT NPs + NIR. **D** Body weight curves of the 4T1 tumor-bearing mice during the treatment. Tumor weight (**E**) and the representative photographs of excised tumors (**F**) of each group. *P < 0.05, **P < 0.01, ***P < 0.001

Next, we observed the PTT triggered by the IR820-SS-CPT NPs on the 4T1 tumor-bearing nude mice under laser irradiation. The female nude mice bearing 4T1 tumor were divided into three groups when the tumors grew to 100 mm^3^: (1) PBS (plus Laser), (2) IR820 (plus Laser), and (3) IR820-SS-CPT NPs (plus Laser). At the same time, the IR images, revealed the temperature elevation, were also recorded by the IR thermal camera. As shown in Fig. 6A, B, after exposing to laser irradiation, the tumor temperature of the mice increased rapidly to 50.5 °C after injecting with IR820-SS-CPT NPs. On the contrary, there was no significant temperature increase for the mice injected with PBS, which was not sufficient to cause any tumor destruction.

In order to investigate the therapeutic efficacy of the combination of photothermal therapy (PTT) with chemotherapy, the growth of tumors was recorded after the mice received IR820-SS-CPT NPs with or without irradiation. The tumor growth curves of each group were calculated and presented in Fig. 6C. Obviously, the tumor size of the control groups treated with PBS grew rapidly. Besides, the average tumor size in the IR820-SS-CPT NPs without laser irradiation group was slightly inhibited. In contrast, with laser irradiation, the IR820-SS-CPT NPs displayed the strongest inhibitory effect on tumor growth (Fig. 6E, F). The average tumor size in this group was smaller than any other ones. This might be attributed to the hyperthermia, which would induce the irreversible injury to cancer cells. Moreover, the CPT, effectively released from the IR820-SS-CPT NPs, would accelerate the process of cancer cell death. Besides, there was no obvious loss of the body weight of the mice, suggesting little systemic toxicity of the prodrug during treatment (Fig. 6D).

Further histological analysis of the tumor and major organs was conducted with H&E staining to evaluate the antitumor effect and the biosafety of this combined chemo-photothermal therapy. The IR820-SS-CPT NPs with laser group caused dobvious karyolysis and lesions in the tumor tissues, confirming the ideal antitumor effect (Additional file 1: Fig. S20). Meanwhile, the major organs of the mice did not show noticeable histopathological lesions. All these results demonstrated that the IR820-SS-CPT NPs could produce excellent chemo-photothermal antitumor effects with few side effects.

## Conclusion

In summary, we have synthesized a novel activatable amphiphilic small molecular prodrug. This prodrug integrated multiple advantages (e.g. GSH-triggered drug release, high therapeutic agent content, and combined chemo-photothermal therapy) into one drug delivery system. Benefiting from the amphiphilicity of IR820-SS-CPT, the IR820-SS-CPT NPs could be achieved by the self-assembly method. In vitro study has confirmed that IR820-SS-CPT NPs displayed stronger cytotoxicity to 4T1 cancer cells under laser irradiation. Specially, the IR820-SS-CPT NPs significantly improved the anti-tumor efficacy through the chemo-photothermal combination therapy in vivo. Therefore, IR820-SS-CPT NPs may provide a charming drug delivery system for chemo-photothermal therapy.

## Materials and methods

### Materials

Camptothecin (CPT), IR-820, 2-hydroxyethyl disulfide (HEDS), 1, 6-hexanediol, triphosgene, 4-nitrophenyl chloroformate, 4-dimethylaminopyridine (DMAP), and other reagents were purchased from Sigma-Aldrich (USA). The solvents anhydrous dimethylformamide (DMF), and anhydrous dichloromethane (DCM) were also acquired from Sigma-Aldrich (USA). Fetal bovine serum (FBS) and Dulbecco’s modified Eagle’s medium (DMEM) were purchased from Gibco. All other reagents and solvents were of analytical or chromatographic grade.

### Synthesis

#### CPT-SS-OH [31, 32]

A solution of CPT (1.0 g, 2.87 mmol), DMAP (1.05 g, 8.61 mmol) and triphosgene (0.32 g, 1.05 mmol) in anhydrous DCM (50 mL) was stirred at room temperature for 1 h, then a mixture of 2-hydroxyethyl disulfide (4.43 g, 28.7 mmol) in 10 mL THF was added at 0 °C. The mixture was stirred overnight under this temperature. After the removal of the solvent by rotary evaporation, the product was purified by column chromatography (silica gel column, DCM: MeOH = 50: 1, v/v) to get a slight yellow solid (0.68 g, yield 44%). ^1^H NMR (600 MHz, DMSO-*d*_6_): *δ*8.70 (s, 1H), 8.17 (d, *J* = 8.4 Hz, 1H), 8.13 (d, *J* = 7.9 Hz, 1H), 7.87 (ddd, *J* = 8.4, 7.0, 1.3 Hz, 1H), 7.70–7.75 (m, 1H), 7.09 (s, 1H), 5.53 (d, *J* = 6.2 Hz, 2H), 5.29 (s, 2H), 4.92 (t, *J* = 5.4 Hz, 1H), 4.29–4.36 (m, 2H), 3.55 (qd, *J* = 6.0, 2.3 Hz, 2H), 2.99 (td, *J* = 6.1, 1.7 Hz, 2H), 2.77 (t, *J* = 6.4 Hz, 2H), 2.19 (dt, *J* = 14.9, 7.2 Hz, 2H), 0.93 (t, *J* = 7.4 Hz, 3H). ^13^C NMR (150 MHz, DMSO-*d*_6_): *δ*167.5, 157.0, 153.3, 152.7, 148.3, 146.7, 145.2, 132.1, 130.9, 130.2, 129.4, 129.0, 128.5, 128.2, 119.6, 94.9, 78.4, 66.9, 66.8, 59.7, 50.8, 41.6, 36.7, 30.8, 8.0. ESI–MS (low resolution, positive mode): *m/z* [M+H]^+^, calculated for C_25_H_25_N_2_O_7_S_2_^+^, 529.1; found 529.1.

#### CPT-SS-LG

A mixture of CPT-SS-OH (200 mg, 0.38 mmol), 4-nitrophenyl chloroformate (115 mg, 0.57 mmol) and Et_3_N (58 mg, 0.57 mmol) in anhydrous DCM (30 mL) was stirred at room temperature for 12 h. After the removal of the solvent by rotary evaporation, the product was purified by column chromatography (silica gel column, DCM: MeOH = 100:1, v/v) to get a yellow solid (195 mg, yield 74%). ^1^H NMR (600 MHz, CDCl_3_): *δ* 8.33 (s, 1H), 8.21–8.13 (m, 3H), 7.87 (d, *J* = 8.1 Hz, 1H), 7.76 (t, *J* = 7.2 Hz, 1H), 7.60 (t, *J* = 7.3 Hz, 1H), 7.34–7.24 (m, 3H), 5.62 (d, *J* = 17.1 Hz, 1H), 5.32 (d, *J* = 17.1 Hz, 1H), 5.22 (d, *J* = 4.8 Hz, 2H), 4.47–4.26 (m, 4H), 2.99–2.84 (m, 4H), 2.23–2.06 (m, 2H), 0.94 (t, *J* = 7.4 Hz, 3H). ^13^C NMR (150 MHz, CDCl_3_): *δ* 166.3, 156.2, 154.4, 152.5, 151.3, 151.2, 147.9, 145.5, 144.6, 144.4, 130.2, 129.7, 128.6, 127.5, 127.2, 127.2, 127.1, 124.3, 120.8, 119.2, 94.9, 77.1, 66.1, 65.7, 65.5, 49.0, 35.8, 35.7, 30.9, 6.6. ESI–MS (low resolution, positive mode): *m/z* [M+H]^+^, calculated for C_32_H_28_N_3_O_11_S_2_^+^, 694.1; found 694.1.

#### IR820-NH_2_ [33]

IR820 (80% dye, 200 mg, 0.19 mmol), 1, 6-hexanediamine (23 mg, 0.2 mmol) and Et_3_N (40 mg, 0.4 mmol) were dissolved in acetonitrile (5 mL). The green solution was stirred and heated in a pre-warmed oil bath at 70 °C for 3 h. The reaction was cooled down and the solvent evaporated under vacuum to dryness. The crude was purified by column chromatography (silica gel column, DCM: MeOH = 10:1 to 4:1, v/v) to afford the IR820-NH_2_ as a blue solid (76 mg, yield 43%). ^1^H NMR (600 MHz, CD_3_OD): *δ* 8.14 (d, *J* = 8.6 Hz, 2H), 7.91–7.89 (m, 4H), 7.87–7.84 (m, 2H), 7.56–7.53 (m, 2H), 7.45 (d, *J* = 8.8 Hz, 2H), 7.37 (t, *J* = 7.3 Hz, 2H), 5.90 (d, *J* = 13.0 Hz, 2H), 4.15–4.06 (m, 4H), 3.84 (t, *J* = 6.8 Hz, 2H), 2.91–2.87 (m, 6H), 2.58 (t, *J* = 6.2 Hz, 4H), 2.00–1.86 (m, 26H), 1.69–1.67 (m, 4H). ^13^C NMR (150 MHz, CD_3_OD): *δ* 169.0, 168.6, 140.7, 131.1, 131.0, 129.9, 129.6, 128.5, 126.9, 123.5, 121.6, 110.1, 67.7, 50.7, 48.2, 46.5, 39.3, 39.2, 38.8, 30.2, 28.7, 27.3, 27.1, 26.9, 26.0, 25.8, 25.7, 25.5, 24.8, 23.5, 22.6, 22.3, 21.6, 13.0, 10.0, 7.8. ESI–MS (low resolution, negative mode): *m/z* [M−Na]^−^, calculated for C_52_H_65_N_4_O_6_S_2_^−^, 905.4; found 905.4.

#### IR820-SS-CPT

CPT-SS-LG (100 mg, 0.14 mmol), IR820-NH_2_ (109 mg, 0.12 mmol) and Et_3_N (40 mg, 0.4 mmol) were dissolved in anhydrous DMF (5 mL). The solution was stirred at room temperature for 24 h under nitrogen atmosphere and protected from the light. Then the solution was poured into 100 mL diethyl ether, collected the solid and the crude product was purified by column chromatography (silica gel column, DCM: MeOH = 8:1 to 5:1, v/v) to give the IR820-SS-CPT as a blue solid (37 mg, yield 18%). ^1^H NMR (600 MHz, DMSO-*d*_6_): *δ* 8.66 (s, 1H), 8.14 (d, *J* = 8.4 Hz, 3H), 8.10 (d, *J* = 8.1 Hz, 1H), 7.95 (dd, *J* = 3.9, 8.5 Hz, 4H), 7.83 (t, *J* = 7.5 Hz, 1H), 7.74–7.72 (m, 2H), 7.70–7.66 (m, 1H), 7.57–7.53 (m, 4H), 7.37 (t, *J* = 7.5 Hz, 2H), 7.07 (s, 1H), 5.85 (d, *J* = 10.5 Hz, 2H), 5.56–5.48 (m, 2H), 5.27 (s, 2H), 4.29 (t, *J* = 5.7 Hz, 2H), 4.14–4.01 (m, 6H), 3.73 (d, *J* = 4.4 Hz, 2H), 3.00–2.93 (m, 4H), 2.82 (t, *J* = 6.2 Hz, 2H), 2.56–2.52 (m, 6H), 2.16 (td, *J* = 7.2, 14.3 Hz, 2H), 1.92–1.85 (m, 12H), 1.84–1.70 (m, 14H), 1.45–1.38 (m, 4H), 1.34–1.30 (m, 2H), 0.91 (t, *J* = 7.3 Hz, 3H). ^13^C NMR (150 MHz, DMSO-*d*_6_) δ 168.8, 167.5, 156.9, 156.3, 153.3, 152.6, 148.3, 146.7, 145.2, 141.1, 138.3, 132.1, 131.1, 130.9, 130.8, 130.3, 130.3, 130.2, 130.1, 129.4, 129.0, 128.5, 128.3, 128.2, 127.7, 123.9, 122.1, 119.6, 111.4, 94.8, 78.4, 66.9, 66.7, 61.9, 58.4, 51.4, 50.8, 49.4, 46.2, 40.7, 40.5, 37.6, 36.6, 30.8, 29.8, 28.2, 26.5, 26.2, 25.2, 23.1, 21.9, 9.1, 8.0. ESI–MS (low resolution, positive mode): *m/z* [M+H]^+^, calculated for C_78_H_88_N_6_NaO_14_S_4_^+^, 1483.5; found 1483.6.

#### IR820-CC-CPT

The non-activatable prodrug IR820-CC-CPT was synthesized by following the same method as IR820-SS-CPT preparation (Scheme 1) using 1,6-hexanediol instead of 2-hydroxyethyl disulfide as the reduction non-cleavable linker. ^1^H NMR (600 MHz, CD_3_OD): *δ* 8.47 (s, 1 H), 8.01 (d, *J* = 8.4 Hz, 3H), 7.93 (d, *J* = 8.1 Hz, 1H), 7.77 (d, *J* = 8.1 Hz, 6H), 7.72 (t, *J* = 7.5 Hz, 1H), 7.60–7.55 (m, 1H), 7.41 (t, *J* = 7.5 Hz, 2H), 7.33 (d, *J* = 8.8 Hz, 2H), 7.26–7.21 (m, 3H), 5.79 (d, *J* = 12.5 Hz, 1H), 5.50 (d, *J* = 16.5 Hz, 1H), 5.35 (d, *J* = 16.7 Hz, 1H), 5.21–5.09 (m, 2H), 4.02–3.90 (m, 6H), 3.74–3.63 (m, 4H), 2.80–2.76 (m, 4H), 2.51–2.47 (m, 4H), 2.12–2.03 (m, 2H), 1.89–1.73 (m, 24H), 1.47–1.30 (m, 8H), 0.92 (t, *J* = 7.3 Hz, 3H). ESI–MS (low resolution, positive mode): *m/z* [M+H]^+^, calculated for C_80_H_92_N_6_NaO_14_S_2_^+^, 1447.6; found 1447.4.

### Characterization of IR820-CPT conjugate

The ^1^H NMR and ^13^C NMR spectra were obtained by a Bruker AV600 Ultrashield spectrometer at 600 and 150 MHz, respectively (BrukerBiospin, Zug, Switzerland). Mass spectra were obtained on AB SCIEX QTRAP 5500. The ultraviolet–visible–near infrared (UV–vis–NIR) absorption spectra were measured on a Varian Cary 500 spectrophotometer. The fluorescence spectra were recorded on a Varian Cary Eclipse fluorescence spectrophotometer.

### Preparation of IR820-SS-CPT NPs

The IR820-SS-CPT NPs were achieved by the dialysis method. Firstly, IR820-SS-CPT (3 mg) was dissolved in DMSO (1 mL). Subsequently, the solution was dialyzed against distilled water for 24 h with frequent exchanges of water. Additionally, the IR820-CC-CPT NPs were prepared with the same procedure, except that the IR820-SS-CPT was replaced by IR820-CC-CPT.

### Characterization of the IR820-SS-CPT NPs

For morphology characterization, the IR820-SS-CPT NPs were observed by using TEM (JEM1400, JEOL, Japan). The size and zeta potential of the nanoparticles were measured by DLS (Malvern Instruments Ltd., Worcestershire, UK).

### In vitro photothermal effect

The NIR laser was applied for treating the IR820-SS-CPT NPs solution for investigating its photothermal effect. The IR820-SS-CPT NPs and free IR820 were handled with 660 nm and 808 nm NIR laser for 5 min (1 W/cm^2^), respectively. The temperature change of the solutions and real-time thermal imaging were recorded by the infrared imaging camera (FLIR A5, FLIR Systems, USA).

### In vitro drug release

The drug release experiment of IR820-SS-CPT NPs in vitro was conducted by using different concentrations of GSH at the temperature of 37 °C. Briefly, 1 mL of IR820-SS-CPT NPs (0.5 mg/mL) was placed into a dialysis bag (MWCO = 1000 Da). The dialysis bag was immersed into the corresponding buffer solutions. At the designed time intervals, 3 mL of the medium was withdrawn, while an equal volume of fresh PBS was added back. The release amount of CPT was calculated by HPLC method.

The actual released CPT in its original form from the GSH treated IR820-SS-CPT NPs was analyzed by liquid chromatography–mass spectrometry (LC–MS). The LC/MS system consists of an analytical high performance liquid chromatography separation module Thermo Scientific UltiMate 3000 HPLC coupled with a Q Exactive mass spectrometer (Thermo Fisher Scientific, USA). Samples were analyzed using a reversed-phase C18 column (Luna C18, 250 mm × 4.6 mm, 5 µm, Phenomenex). The solvent system was composed of two solutions: solution A [95% H_2_O, 5% acetonitrile (ACN) and 0.1% formic acid (FA)] and solution B (5% H_2_O and 95% ACN). The 20 min gradient LC separation included 3 steps: 90% solvent A in 0–5 min (isocratic); 90–5% solvent A for 5–15 min (linear); 5% solvent A for 15–20 min (isocratic).

### In vitro cellular uptake and intracellular localization observation

4T1 cells were seeded into 6-well plates at a density of 2.0 × 10^4^ cells per well for 24 h incubation, and then treated with free IR820 or IR820-SS-CPT NPs (10 µM) for 0.5, 2, and 4 h at 37 °C. After incubation, the cells were washed thrice with PBS and then fixed by a formalin solution at − 20 °C for 20 min. Subsequently, the cells were washed three times with PBS and then were imaged using a Leica TCS SP5 confocal laser scanning microscopy with excitation at 633 nm.

### Flow cytometry measurement

Qualitative cellular uptake of IR820-SS-CPT NPs was observed by flow cytometry analysis. 4T1 cells were seeded at a density of 2 × 10^5^ cells per well into 6-well plates and further cultured for 24 h. The cells were incubated with IR820 or IR820-SS-CPT NPs for 0.5, 2, and 4 h. Then, the cells were rinsed with PBS for three times. After that, the cells were detached with trypsin/EDTA, suspended in PBS with 10% FBS, harvested by centrifugation at 1000 rpm for 5 min at 4 °C. The data were detected on an Attune NxT flow cytometer (Thermo Fisher Scientific, USA) and the data were analyzed using FlowJo software [34].

To reveal the possible uptake mechanisms of IR820-SS-CPT NPs by 4T1 cells, the uptake study was performed. 4T1 cells were pre-incubated with different inhibitors for 30 min at 37 °C: chlorpromazine, nystatin, and amiloride. The chlorpromazine, nystatin, and amiloride played the role of clathrin-mediated endocytosis inhibitor, caveolae-mediated endocytosis inhibitor, and macropinocytosis inhibitor respectively. The control cells were treated with the DMEM medium without inhibitors. Then, the cells were cultured with IR820-SS-CPT NPs for another 2 h in the presence of these endocytic inhibitors to study the possible mechanisms involved in the uptake of IR820-SS-CPT NPs. The relative uptake index (RUI) was investigated by the following formula:

$${\text{RUI}} = {\text{Fs}}/{\text{Fc}} \times 100\% ,$$

where Fc was the fluorescence intensity of the control and Fs was the fluorescence intensity of IR820-SS-CPT NPs treated with different endocytic inhibitors.

### In vitro cytotoxicity assay

In vitro cytotoxicity evaluations of IR820-SS-CPT NPs against 4T1 and human normal liver cells (LO2) cells were investigated using MTT assays. The 4T1 and LO2 cells were cultured in 96-well plates and then incubated at 37 °C with 5% CO_2_ overnight. Then, various samples were added to the culture wells with incubation. After 6 h of incubation, the cultural medium was replaced. Then, the cells of the group were irradiated with laser at a power of 1.0 W/cm^2^ for 5 min, while other groups without any laser irradiation treatment were used as controls. After incubation for 36 h, MTT solution was added in the medium, following with incubation for another 4 h. Then, DMSO was added to thoroughly dissolve the crystals. Finally, every sample was measured using a microplate reader.

### In vivo pharmacokinetic (PK) analysis

Healthy SD rats (180–200 g) were divided into 2 groups randomly (n = 3), which were intravenously injected with CPT or IR820-SS-CPT NPs. Blood samples were collected into heparinized tubes at different time intervals. They were centrifuged at 4000 rpm for 15 min at 4 °C, and 100 μL of its supernate plasma was mixed with 200 μL acetonitrile to precipitate all the proteins. After centrifuging at 10,000 rpm for 10 min, the organic layer was collected. And 20 μL of the acetonitrile solution was tested by LC–MS to determine the drug concentration in each plasma sample. Pharmacokinetic data analyses were conducted using a noncompartmental analysis model (DAS 3.2.8, T.C.M., Shanghai, China).

### In vivo imaging and biodistribution

When the tumor volume reached about 100 mm^3^, the 4T1 tumor-bearing nude mice were randomly divided into 2 groups. Then, IR820 (5 mg/kg) and IR820-SS-CPT NPs were injected by intravenous administration respectively. In vivo images were obtained by IVIS Lumina imaging system (Caliper, USA) at different time points after injection (3, 6, 12, 24 h). Finally, all the major organs and tumors were dissected, which were collected for semi-quantitative analysis and ex vivo fluorescence imaging.

### In vivo PA imaging

4T1 tumor-bearing nude mice were respectively injected with the IR820 and IR820-SS-CPT NPs by intravenous administration. At different time points, the PA signals of the tumors were obtained by Vevo LAZR-X multimodal imaging system (FUJIFILM VisualSonics, Canada).

### In vivo photothermal effect of IR820-SS-CPT NPs

The female mice bearing 4T1 tumor were divided into three groups when the tumors grew to 100 mm^3^: (1) PBS (plus Laser), (2) IR820 (plus Laser), and (3) IR820-SS-CPT NPs (plus Laser). Then, the tumor sites of the IR820 treated group were illuminated with NIR laser (808 nm, 1.0 W/cm^2^) for 5 min, while the tumor sites of the IR820-SS-CPT NPs treated mice were illuminated with NIR laser (660 nm, 1.0 W/cm^2^) for 5 min. Meanwhile, the temperature images of the mice were collected by a thermal imaging instrument.

### In vivo combinational therapy of IR820-SS-CPT NPs

The female 4T1 tumor bearing nude mice were divided into six groups (n = 3) when the tumors grew to 100 mm^3^: (a) PBS, (b) IR820, (c) IR820 (plus Laser), (d) CPT, (e) IR820-SS-CPT NPs and (f) IR820-SS-CPT NPs (plus Laser). Then, the mice of group c were illuminated with NIR laser (808 nm, 1.0 W/cm^2^) for 5 min at 12 h post-injection, while the group f were illuminated with NIR laser (660 nm, 1.0 W/cm^2^) for 5 min at 12 h post-injection. Different formulation was injected by intravenous administration in each group every 3 days for 15 days. The tumor volume (V) and body weight of each group were monitored. The tumor volume was counted by “V = longest diameters × shortest diameters^2^/2”.

The tumors and organs of each group were collected at the end of the experiment (all the mice were euthanized), which were fixed with 10% formaldehyde solution. Lastly, all of them were examined by H&E staining for histological analysis.

### Statistical analysis

Data were presented as means ± standard errors. All the statistical analyses were performed using Student’s t-test. Differences were considered statistically significant at a level of p < 0.05 and very significant when p < 0.01.

## Supplementary Information

**Additional file 1: Figure S1.** ^1^H-NMR spectrum of CPT-SS-OH in DMSO-*d*_*6*_. **Figure S2.** ^13^C-NMR spectrum of CPT-SS-OH in DMSO-*d*_*6*_. **Figure S3.** ^1^H-NMR spectrum of CPT-SS-LG in CDCl_3_. **Figure S4.** ^13^C-NMR spectrum of CPT-SS-LG in CDCl_3_. **Figure S5.** ^1^H-NMR spectrum of IR820-NH_2_ in CD_3_OD. **Figure S6.** ^13^C-NMR spectrum of IR820-NH_2_ in CD_3_OD. **Figure S7.** ^1^H-NMR spectrum of IR820-SS-CPT in DMSO-*d*_*6*_. **Figure S8.** ^13^C-NMR spectrum of IR820-SS-CPT in DMSO-*d*_*6*_. **Figure S9.** ESI–MS spectrum of IR820-SS-CPT. **Figure S10.** ^1^H-NMR spectrum of CPT-CC-OH in CDCl_3_. **Figure S11.** ^1^H-NMR spectrum of CPT-CC-LG in CDCl_3_. **Figure S12.** ^1^H-NMR spectrum of IR820-CC-CPT in CD_3_OD. **Figure S13.** ESI–MS spectrum of IR820-CC-CPT. **Figure S14.** UV–vis–NIR absorbance spectra of IR820, IR820-SS-CPT, and CPT in methanol. **Figure S15.** Stability of the hydrodynamic particle size of the IR820-SS-CPT NPs. **Figure S16.** Fluorescence spectra of IR820-SS-CPT NPs (10 μM) incubated with or without 10 mM GSH for 2 h in PBS. **Figure S17.** Relative fluorescent intensity of IR820-SS-CPT NPs internalized by 4T1 cells treated with PBS, chlorpromazine, nystatin, amiloride at 37 °C, and PBS at 4 °C using flow cytometry analysis. **Figure S18.** Relative viability of LO2 cells treated with various concentrations of CPT and IR820-SS-CPT NPs for 36 h. Error bars indicate SD (n = 3). **Figure S19.** In vivo pharmacokinetics profiles of IR820-SS-CPT NPs and free CPT in Sprague–Dawley (SD) rats. Error bars indicate SD (n = 3). **Figure S20.** Representative H&E staining of the major organs and tumors of the mice treated with PBS and IR820-SS-CPT NPs + NIR.

## Data Availability

All data generated or analyzed during this study are included in this published article and its Additional file.

## Abbreviations

CPT: Camptothecin
PTT: Photothermal therapy
NIR: Near-infrared
ADDC: Amphiphilic drug–drug conjugate
NPs: Nanoparticles
GSH: Glutathione
DLS: Dynamic light scattering
TEM: Transmission electron microscopy
PDI: Polydispersity index
LSCM: Laser scanning confocal microscopy
NS: Normal saline
DMAP: 4-Dimethylaminopyridine
DMF: Dimethylformamide
DCM: Dichloromethane
DMSO: Dimethyl sulfoxide
MTT: Methylthiazolydiphenyltetrazolium bromide
