# Supplementary material for: Carrier-free nanoparticles of camptothecin prodrug for chemo-photothermal therapy: the making, in vitro and in vivo testing
Source: J Nanobiotechnology. 2021 Oct 30;19:350. doi: 10.1186/s12951-021-01093-y (PMC8557616; doi:10.1186/s12951-021-01093-y)
Supplement: Supplementary file 1 — Additional file 1: Figure S1. 1H-NMR spectrum of CPT-SS-OH in DMSO-d6. Figure S2. 13C-NMR spectrum of CPT-SS-OH in DMSO-d6. Figure S3. 1H-NMR spectrum of CPT-SS-LG in CDCl3. Figure S4. 13C-NMR spectrum of CPT-SS-LG in CDCl3. Figure S5. 1H-NMR spectrum of IR820-NH2 in CD3OD. Figure S6. 13C-NMR spectrum of IR820-NH2 in CD3OD. Figure S7. 1H-NMR spectrum of IR820-SS-CPT in DMSO-d6. Figure S8. 13C-NMR spectrum of IR820-SS-CPT in DMSO-d6. Figure S9. ESI–MS spectrum of IR820-SS-CPT. Figure S10. 1H-NMR spectrum of CPT-CC-OH in CDCl3. Figure S11. 1H-NMR spectrum of CPT-CC-LG in CDCl3. Figure S12. 1H-NMR spectrum of IR820-CC-CPT in CD3OD. Figure S13. ESI–MS spectrum of IR820-CC-CPT. Figure S14. UV–vis–NIR absorbance spectra of IR820, IR820-SS-CPT, and CPT in methanol. Figure S15. Stability of the hydrodynamic particle size of the IR820-SS-CPT NPs. Figure S16. Fluorescence spectra of IR820-SS-CPT NPs (10 μM) incubated with or without 10 mM GSH for 2 h in PBS. Figure S17. Relative fluorescent intensity of IR820-SS-CPT NPs internalized by 4T1 cells treated with PBS, chlorpromazine, nystatin, amiloride at 37 °C, and PBS at 4 °C using flow cytometry analysis. Figure S18. Relative viability of LO2 cells treated with various concentrations of CPT and IR820-SS-CPT NPs for 36 h. Error bars indicate SD (n = 3). Figure S19. In vivo pharmacokinetics profiles of IR820-SS-CPT NPs and free CPT in Sprague–Dawley (SD) rats. Error bars indicate SD (n = 3). Figure S20. Representative H&E staining of the major organs and tumors of the mice treated with PBS and IR820-SS-CPT NPs + NIR. [file 12951_2021_1093_MOESM1_ESM.docx]

**Additional file 1**

**Carrier-free nanoparticles of camptothecin prodrug for** **chemo-photothermal therapy-The making, in vitro and in vivo testing**

Mingtao Ao^7#^, Fei Yu^1, 6^*^#^, Yixiang Li^1^, Mengya Zhong^3^, Yonghe Tang^6^, Hua Yang^1^, Xiaojing Wu^5^, Yifan Zhuang^3^, Huiyun Wang^4^*, Xiaolian Sun^5^*, Xuehui Hong^3^*, Xiao Dong Chen ^2^*

^1^Medical College, Guangxi University, Nanning 530004, China.

^2^ Suzhou Key Lab of Green Chemical Engineering, School of Chemical and Environmental Engineering, College of Chemistry, Chemical Engineering and Materials Science, Soochow University, Suzhou 215123, China.

^3^Department of Gastrointestinal Surgery, Zhongshan Hospital of Xiamen University, Xiamen 361005, China.

^4^ Department of Pharmacy, Jining Medical College, Sunshine City 276826, China.

^5^ State Key Laboratory of Natural Medicines, Key Laboratory of Drug Quality Control and Pharmacovigilance, Department of Pharmaceutical Analysis, China Pharmaceutical University, Nanjing 210009, China.

^6^Guangxi Key Laboratory of Electrochemical Energy Materials, Guangxi University, Nanning 530004, China.

^7^School of Pharmaceutical Sciences, Xiamen University, Xiamen, 361102, China.


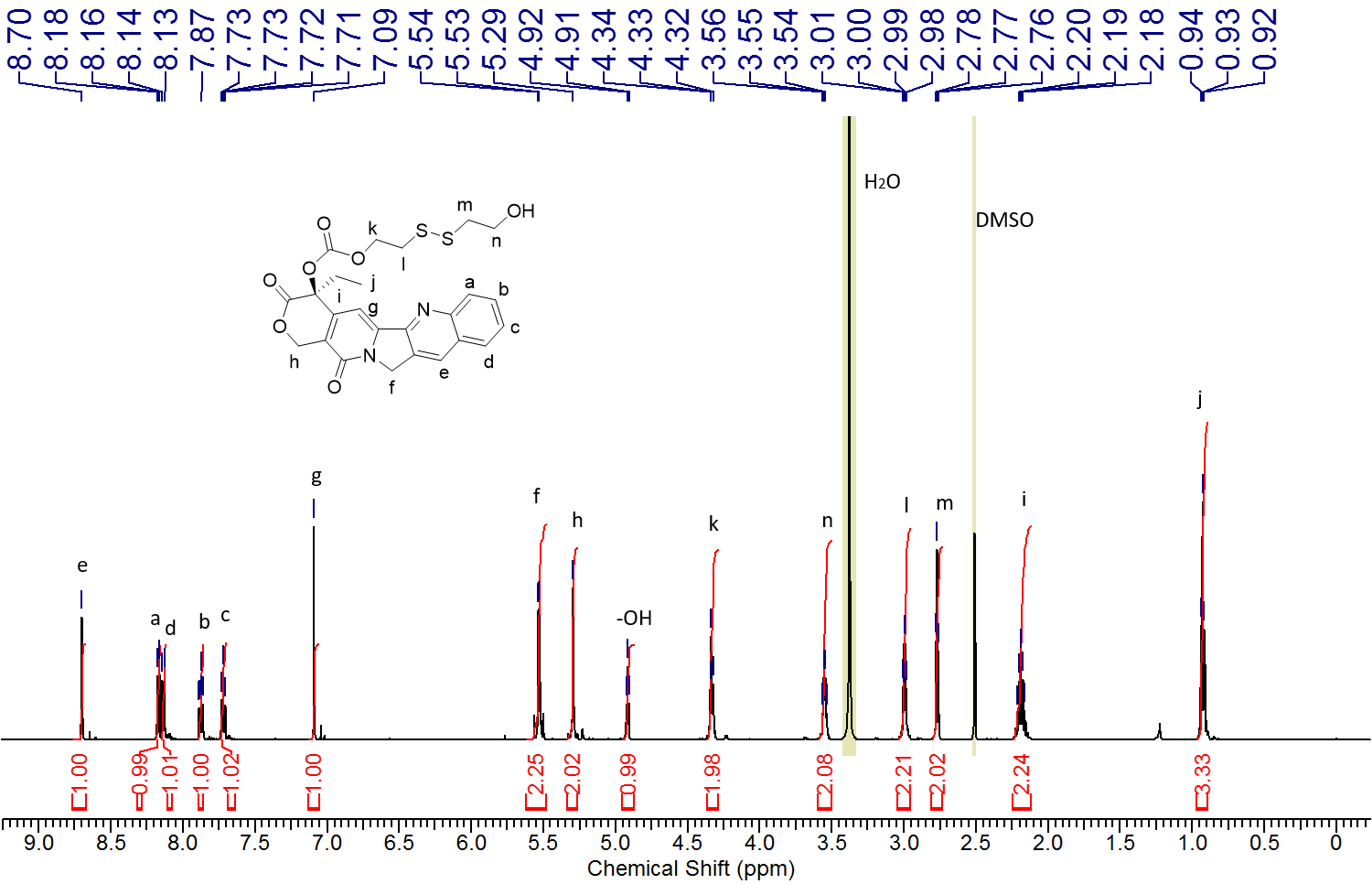


**Figure S1.** ^1^H-NMR spectrum of CPT-SS-OH in DMSO-*d_6_*


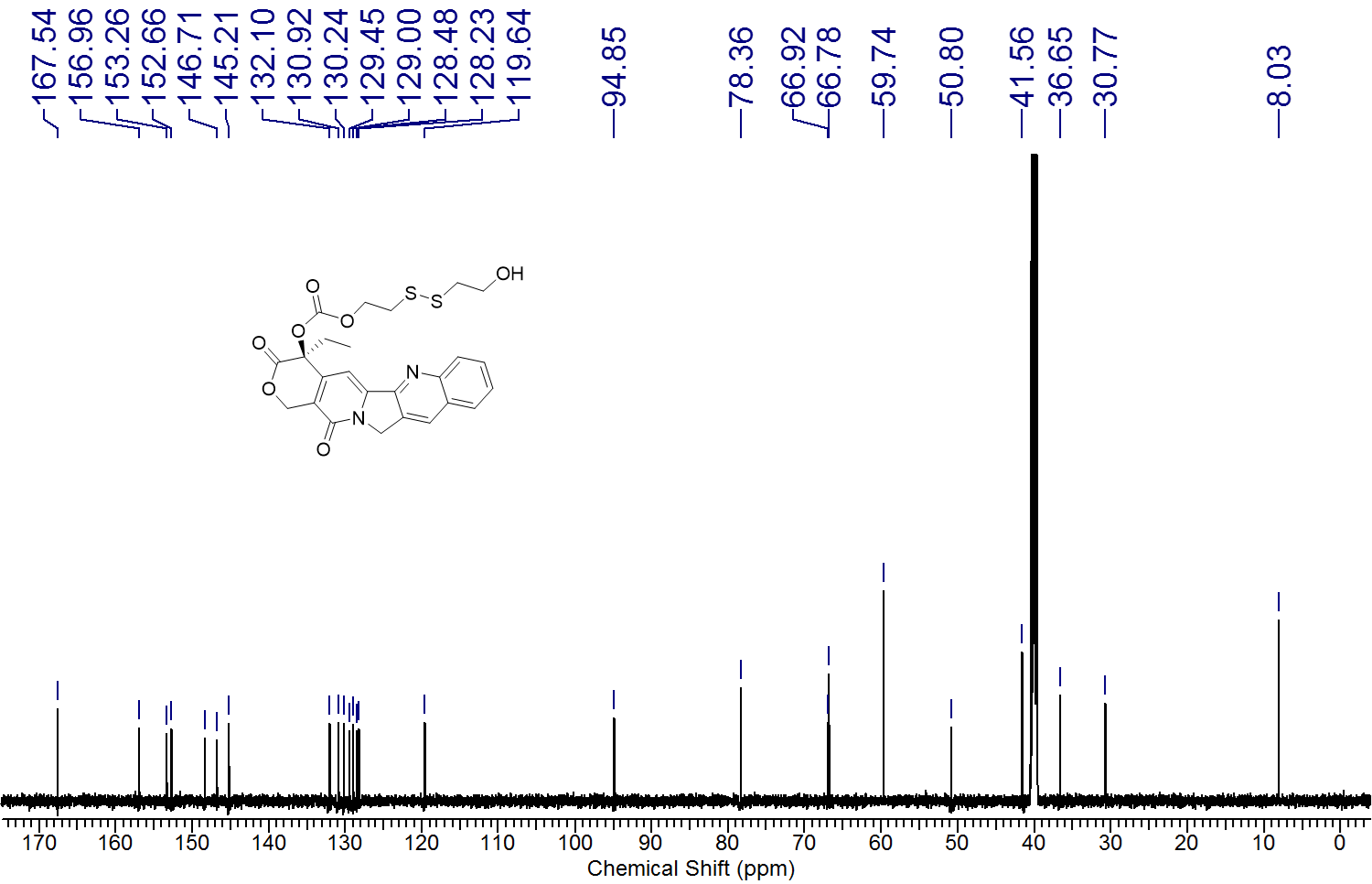


**Figure S2.** ^13^C-NMR spectrum of CPT-SS-OH in DMSO-*d_6_*


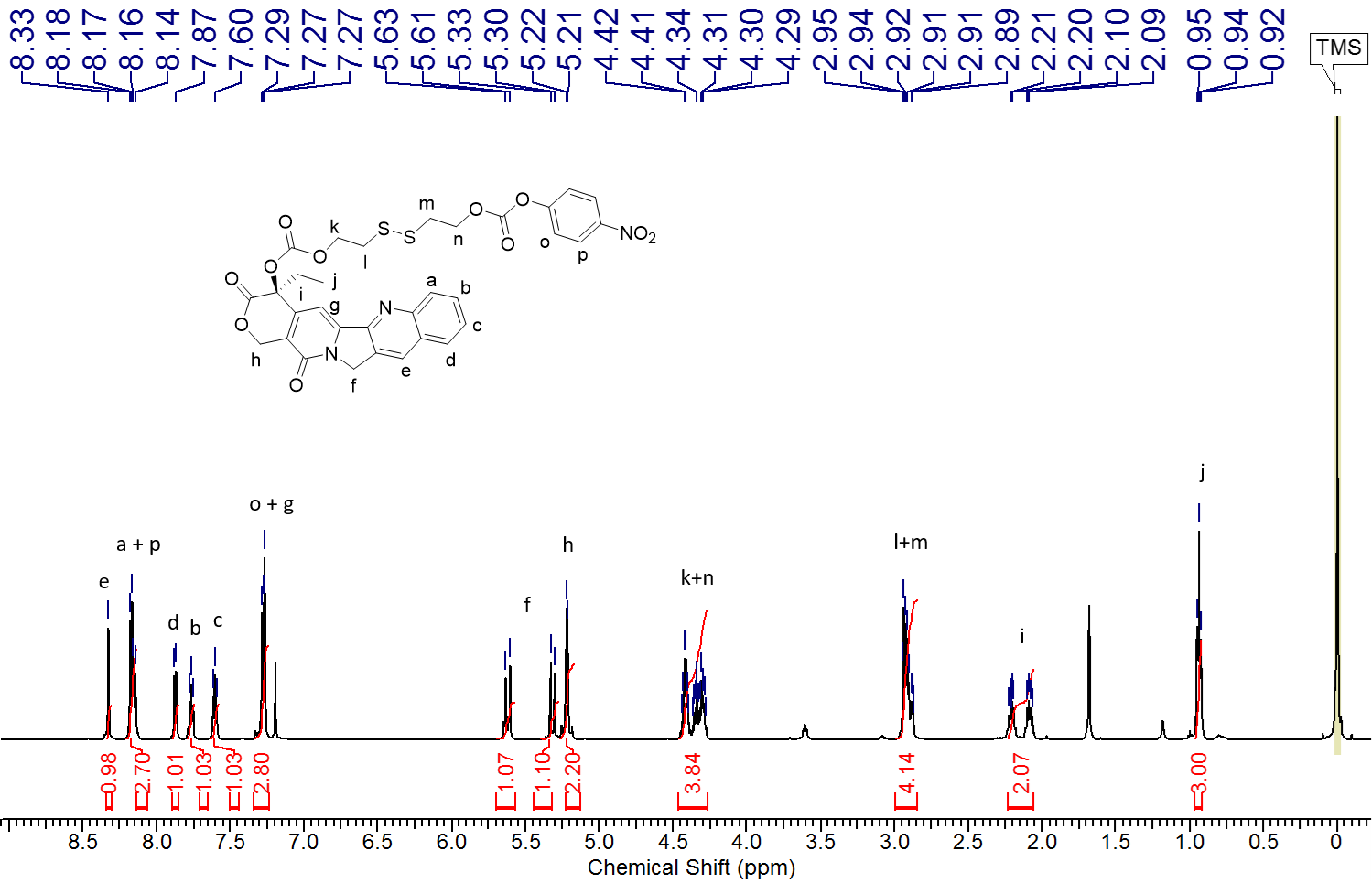


**Figure S3.** ^1^H-NMR spectrum of CPT-SS-LG in CDCl_3_


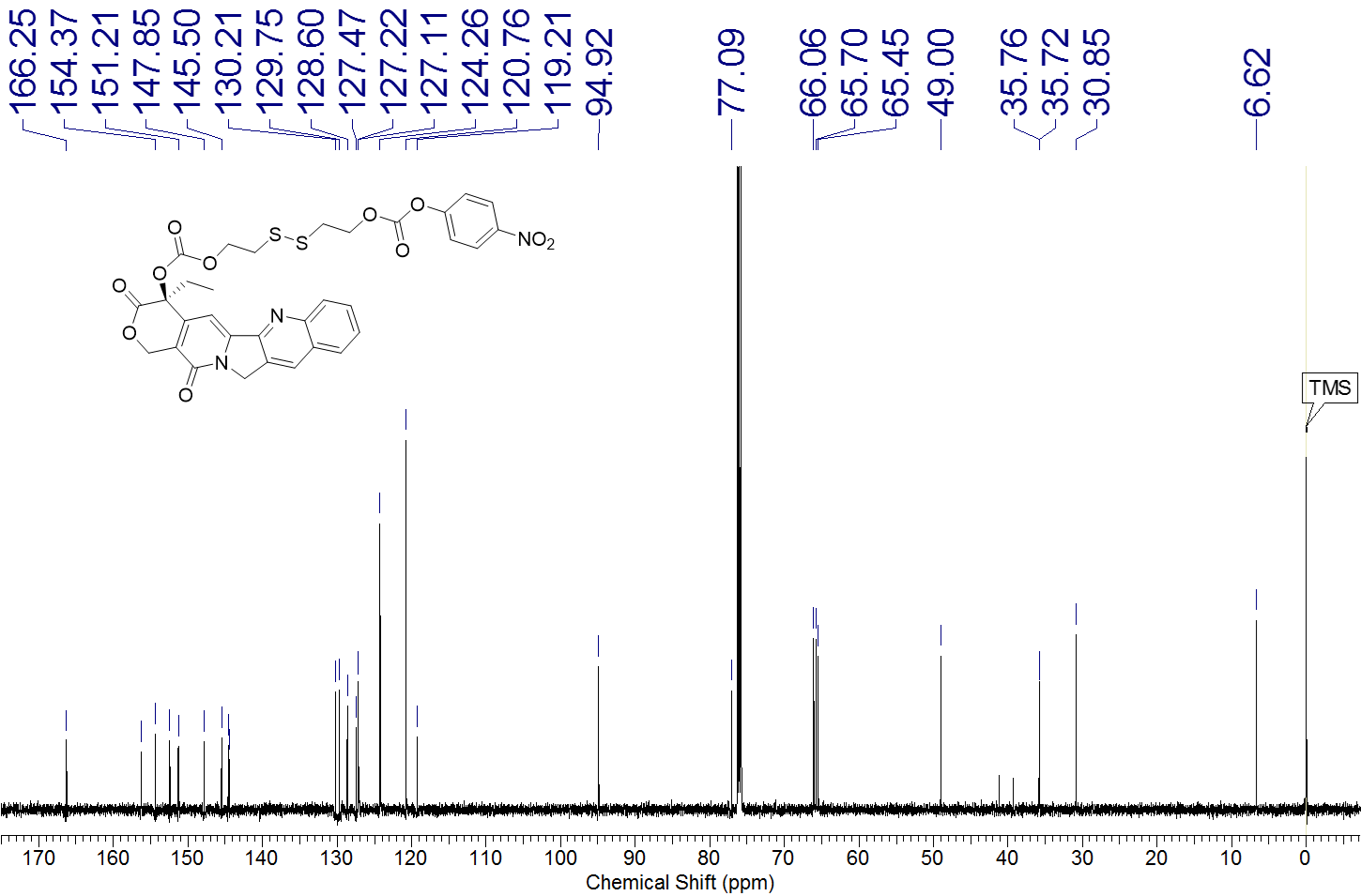


**Figure S4.** ^13^C-NMR spectrum of CPT-SS-LG in CDCl_3_


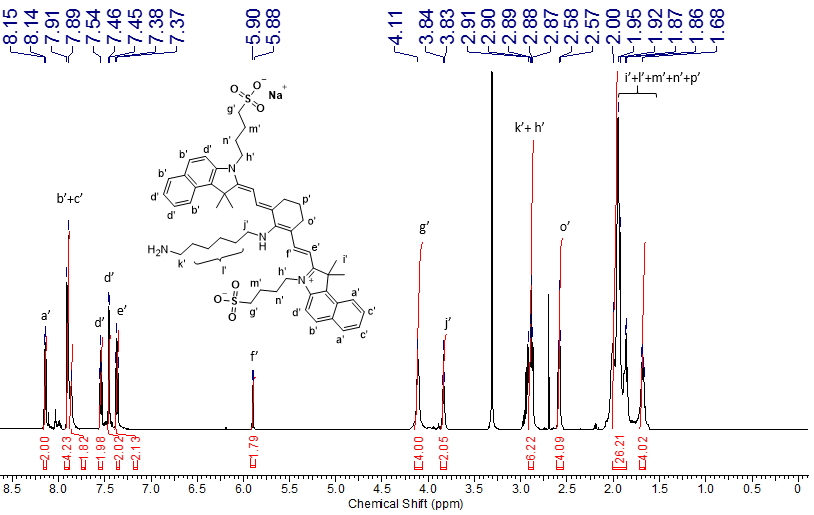


**Figure S5.** ^1^H-NMR spectrum of IR820-NH_2_ in CD_3_OD


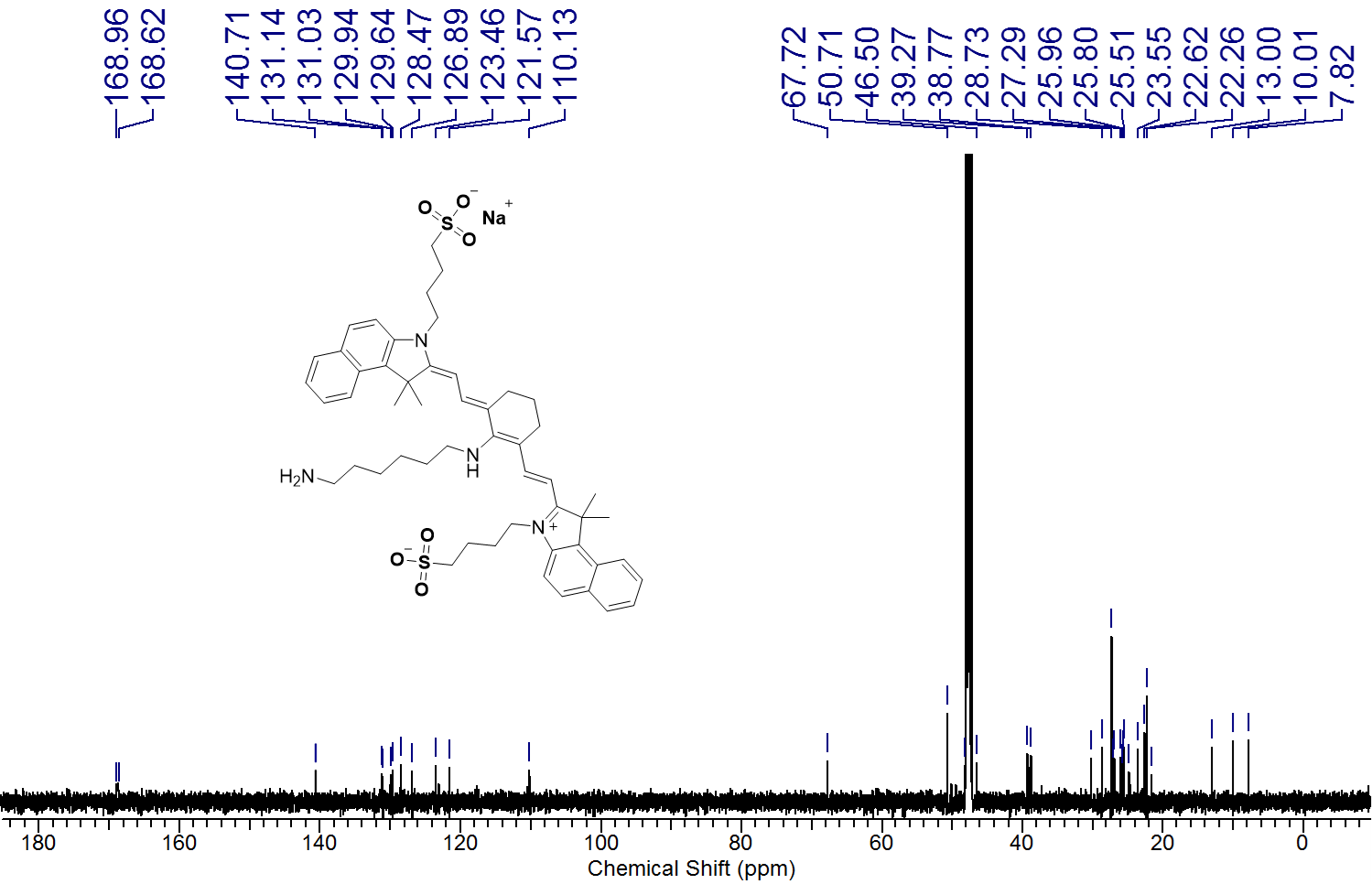


**Figure S6.** ^13^C-NMR spectrum of IR820-NH_2_ in CD_3_OD


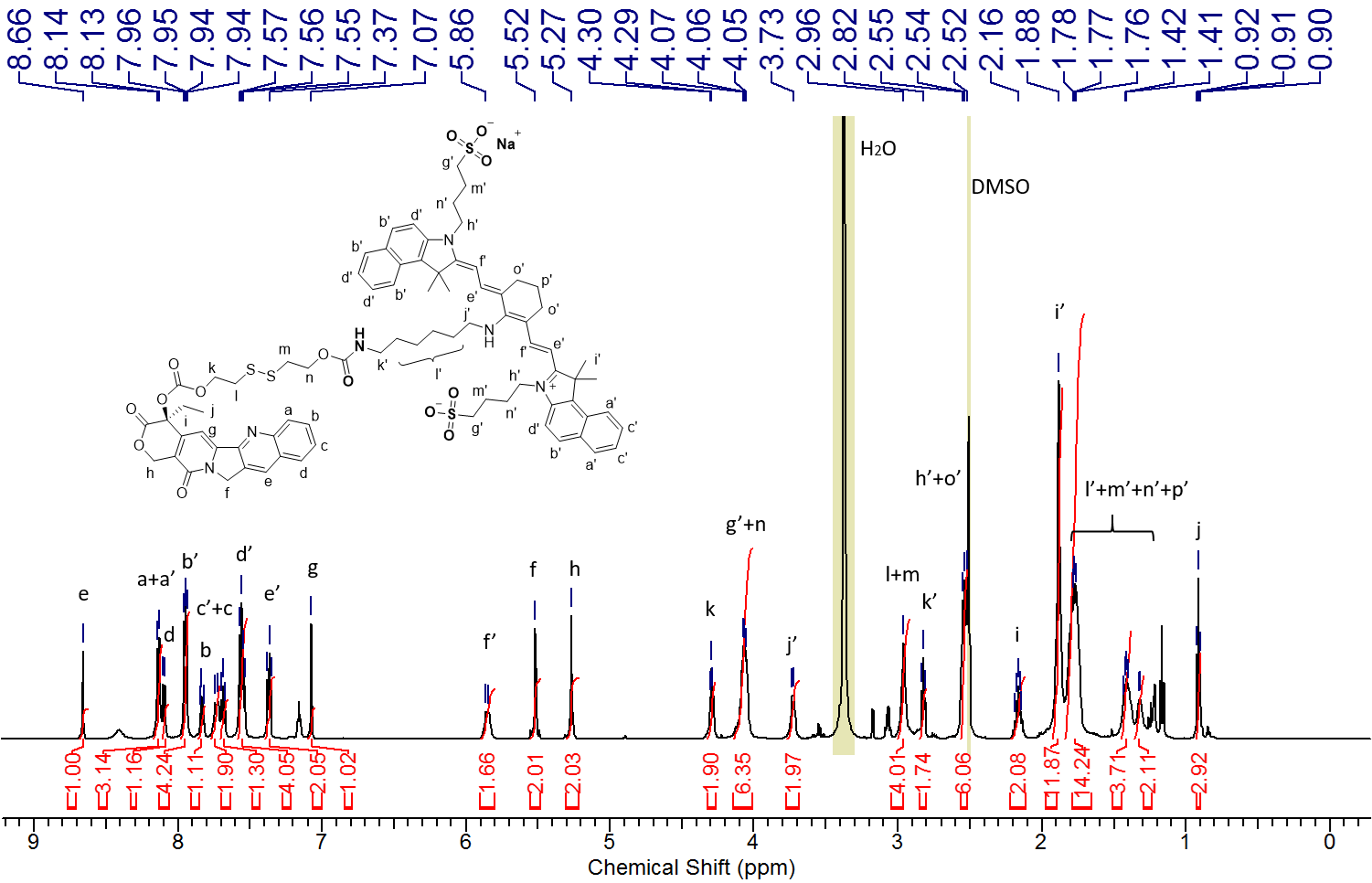


**Figure S7.** ^1^H-NMR spectrum of IR820-SS-CPT in DMSO-*d_6_*


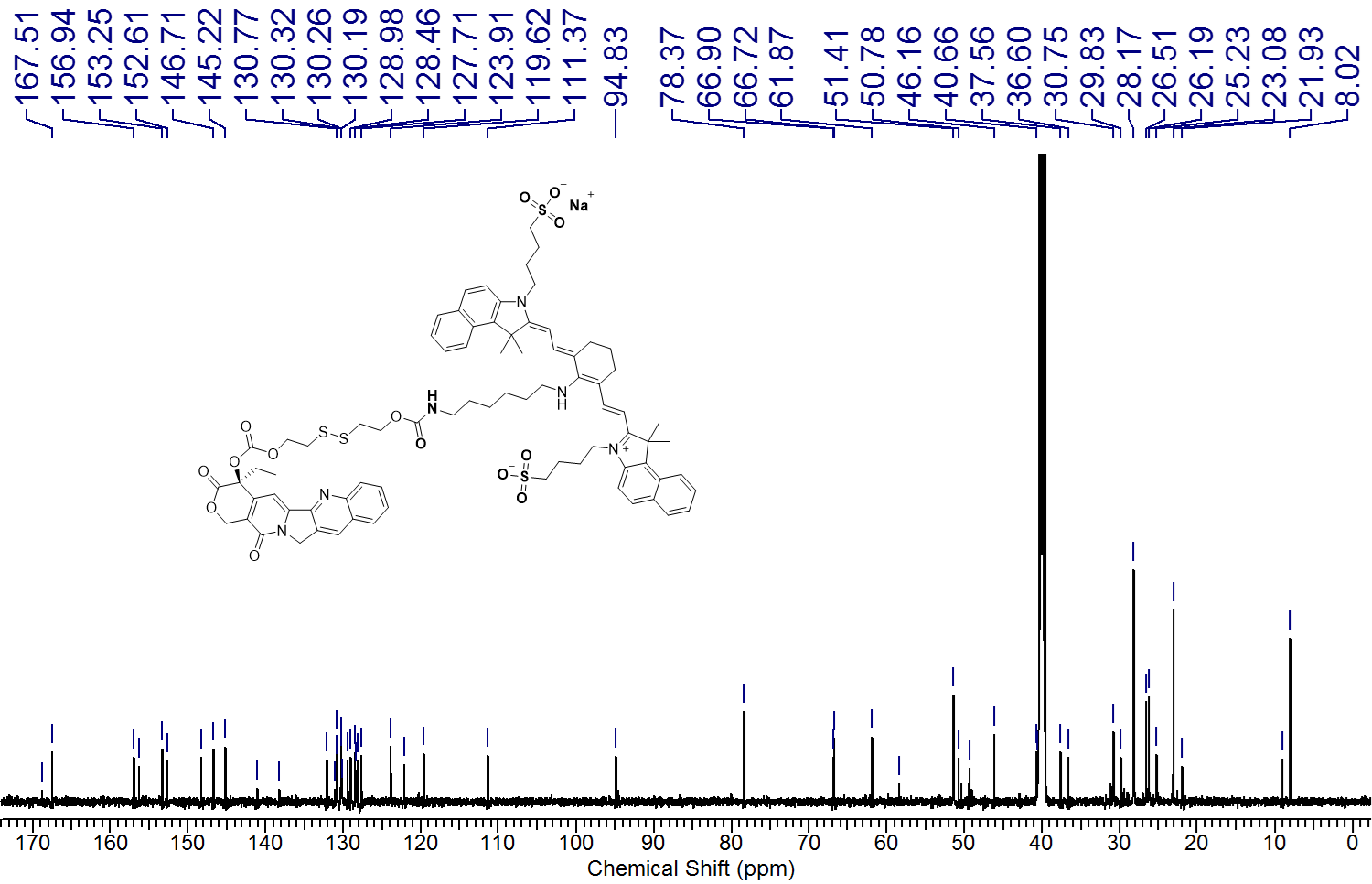


**Figure S8.** ^13^C-NMR spectrum of IR820-SS-CPT in DMSO-*d_6_*


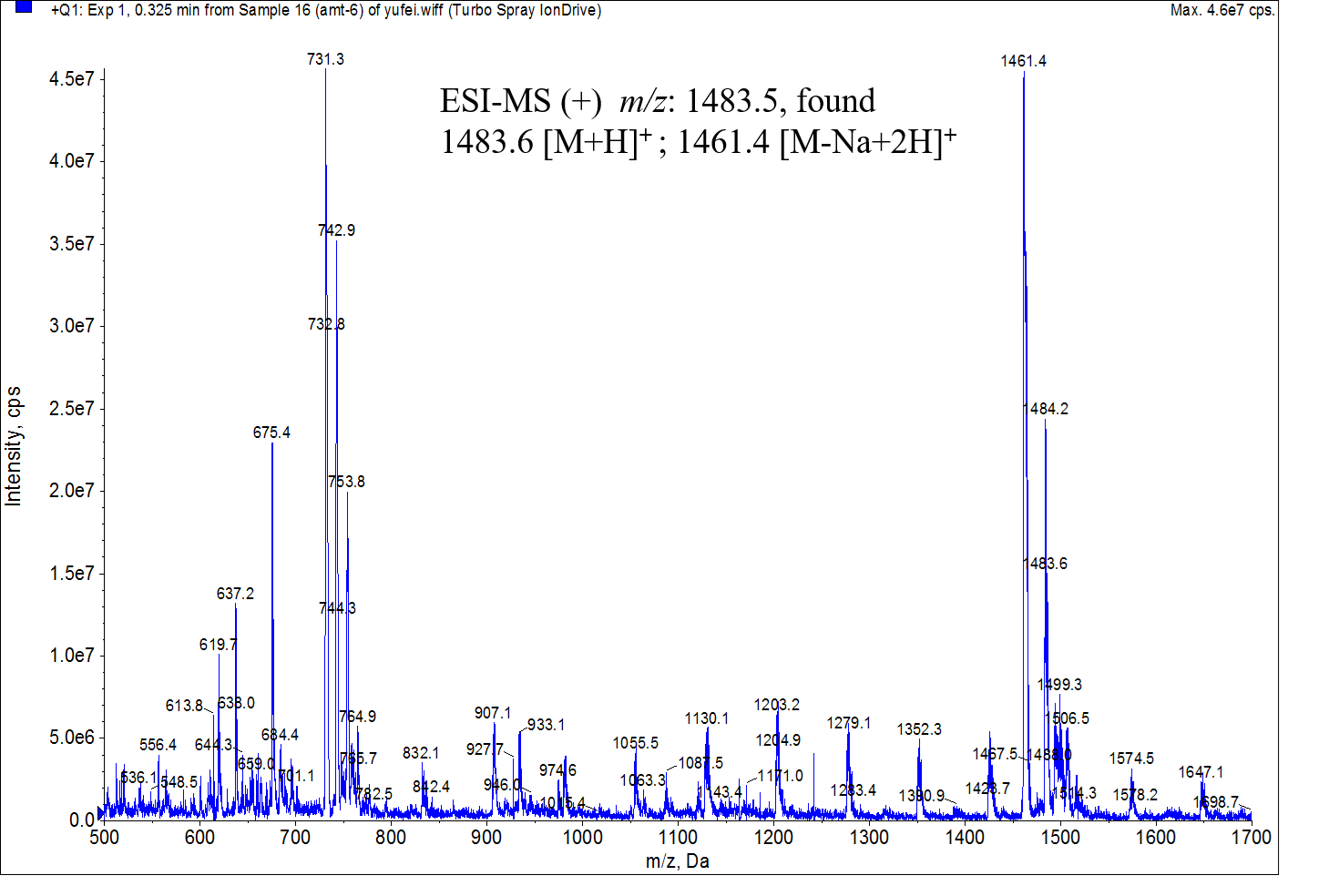


**Figure S9.** ESI-MS spectrum of IR820-SS-CPT


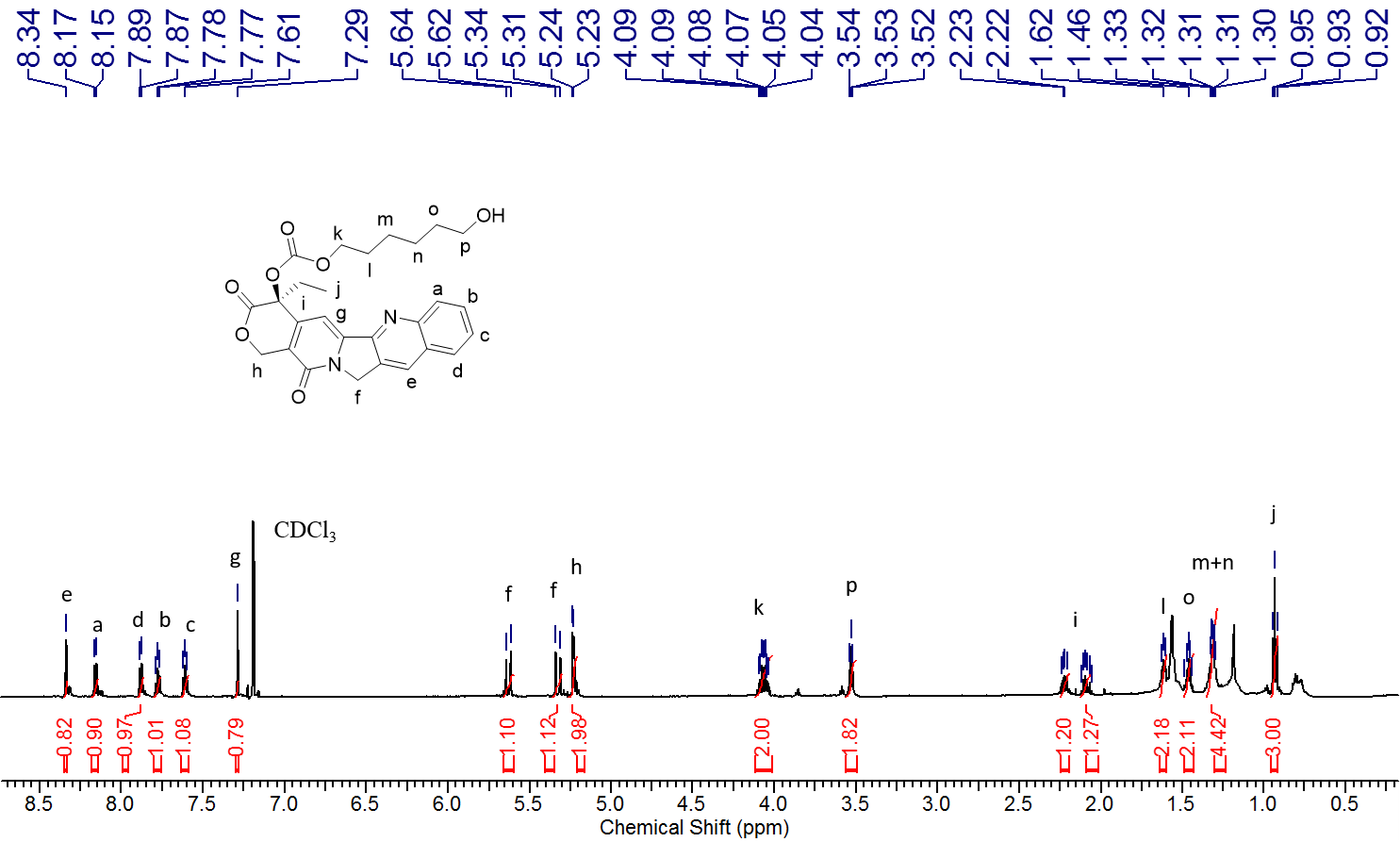


**Figure S10.** ^1^H-NMR spectrum of CPT-CC-OH in CDCl_3_


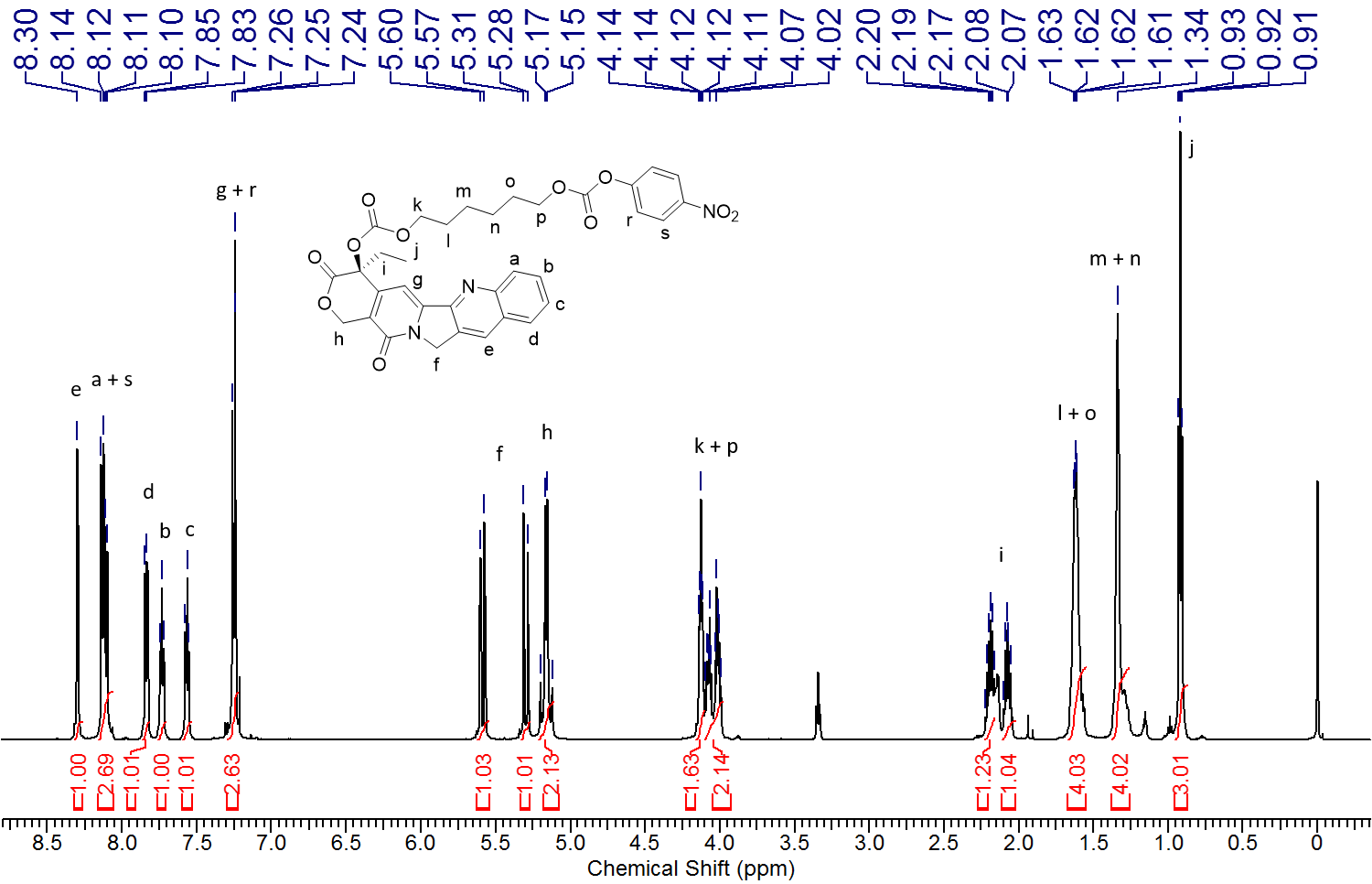


**Figure S11.** ^1^H-NMR spectrum of CPT-CC-LG in CDCl_3_


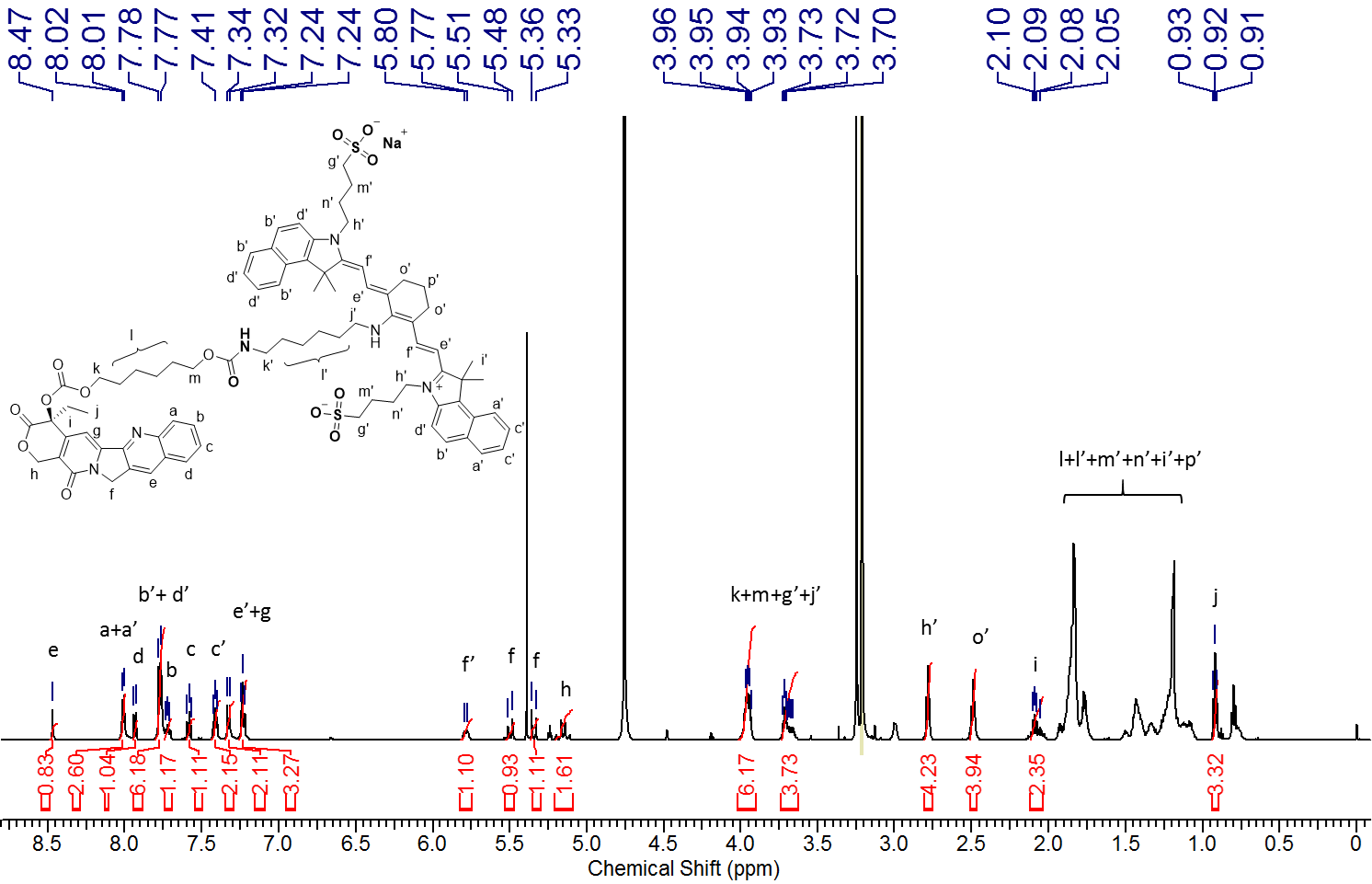


**Figure S12.** ^1^H-NMR spectrum of IR820-CC-CPT in CD_3_OD


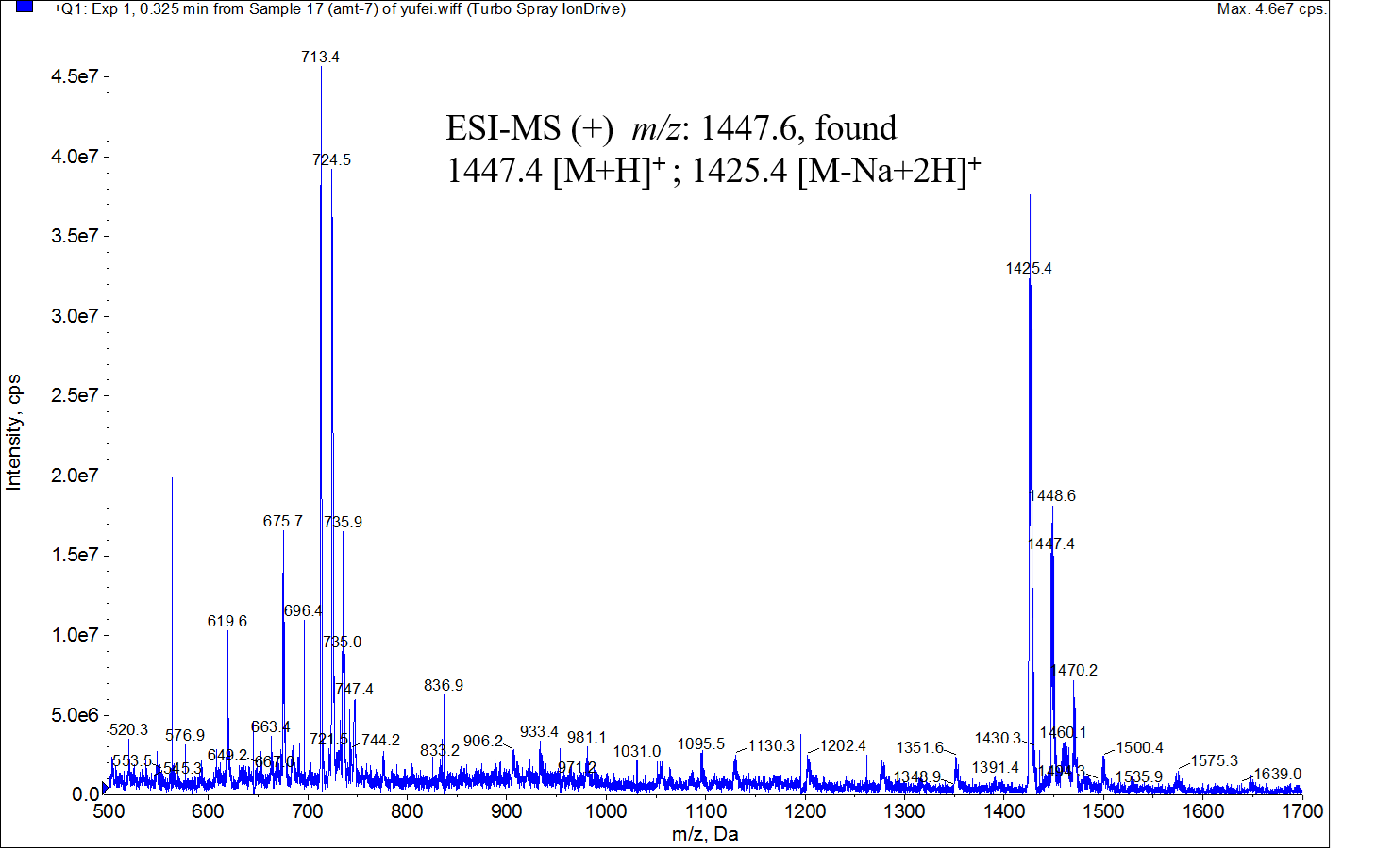


**Figure S13.** ESI-MS spectrum of IR820-CC-CPT.


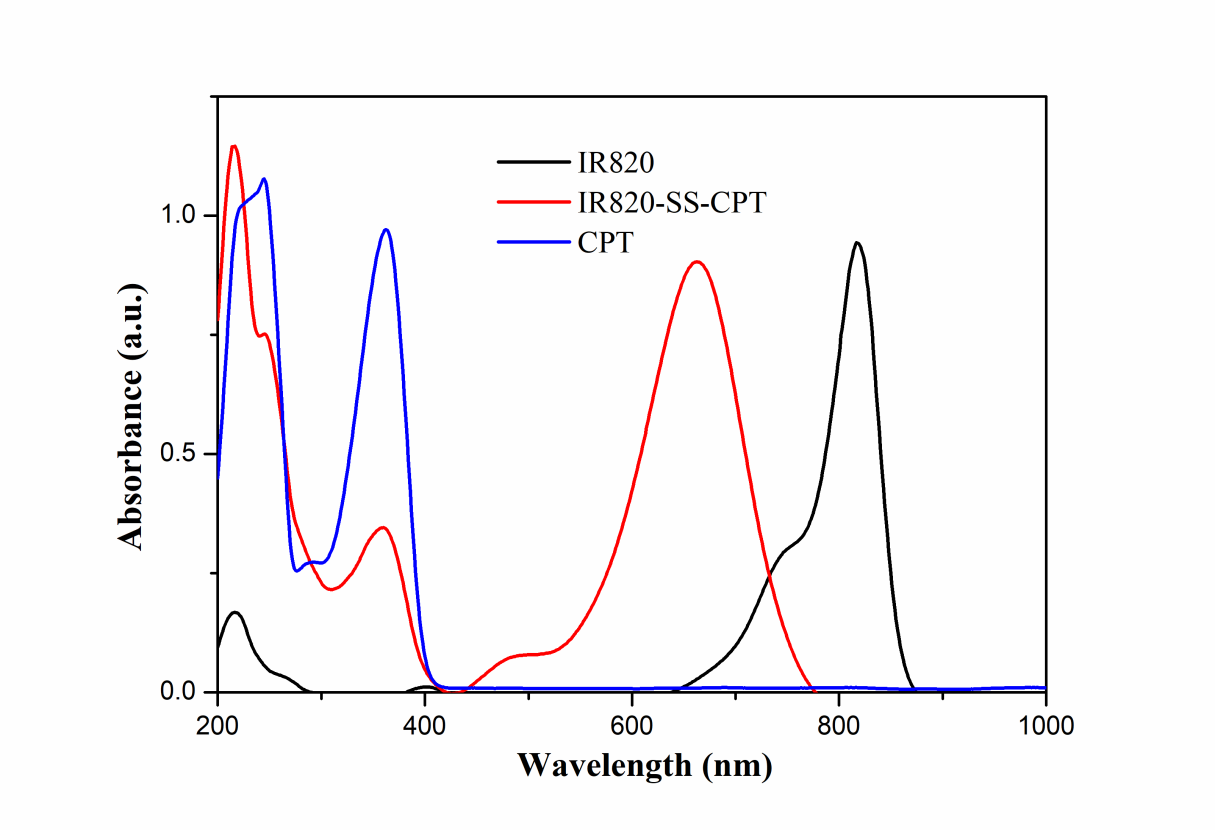


**Figure S14.** UV-vis-NIR absorbance spectra of IR820, IR820-SS-CPT, and CPT in methanol.


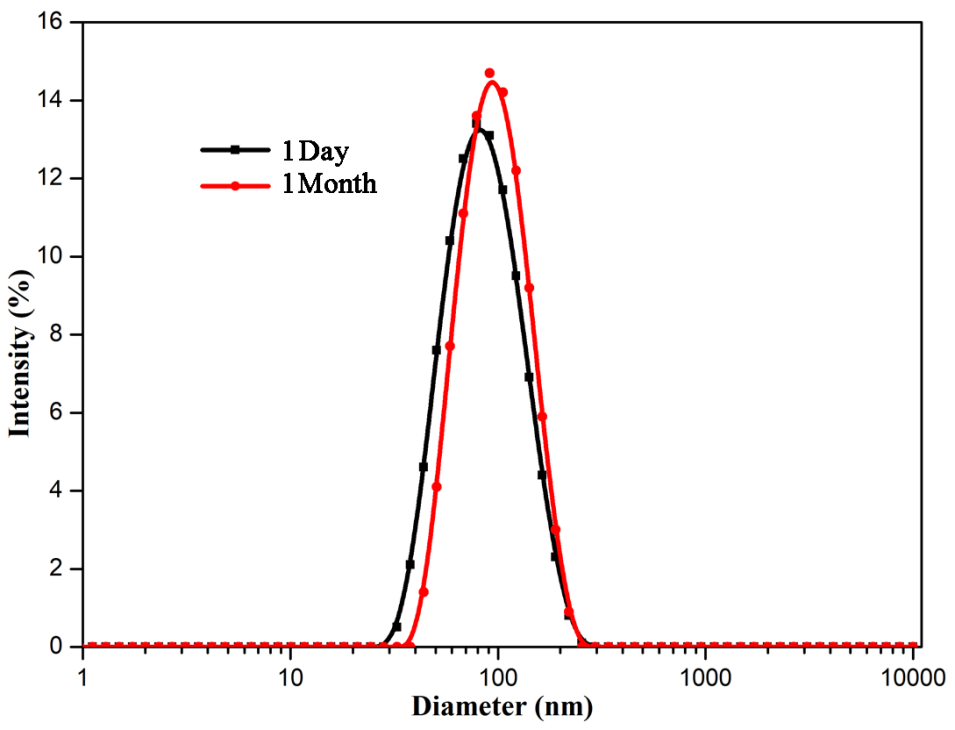


**Figure S15.** Stability of the hydrodynamic particle size of the IR820-SS-CPT NPs.


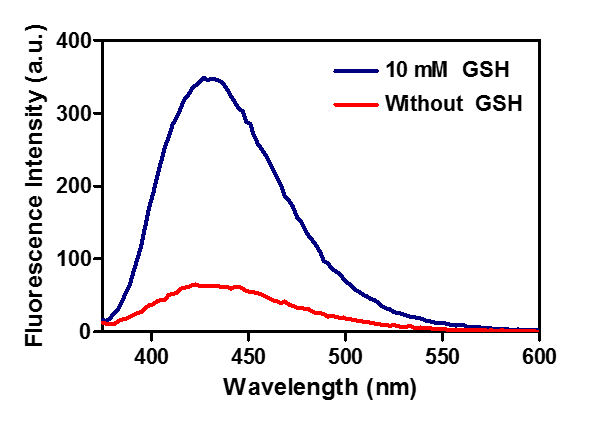


**Figure S16.** Fluorescence spectra of IR820-SS-CPT NPs (10 μM) incubated with or without 10 mM GSH for 2 h in PBS.

**Figure S17.** Relative fluorescent intensity of IR820-SS-CPT NPs internalized by 4T1 cells treated with PBS, chlorpromazine, nystatin, amiloride at 37 ℃, and PBS at 4 ℃ using flow cytometry analysis.

**Figure S18.** Relative viability of LO2 cells treated with various concentrations of CPT and IR820-SS-CPT NPs for 36 h. Error bars indicate SD (n = 3).

**Figure S19.** In vivo pharmacokinetics profiles of IR820-SS-CPT NPs and free CPT in Sprague-Dawley (SD) rats. Error bars indicate SD (n = 3).


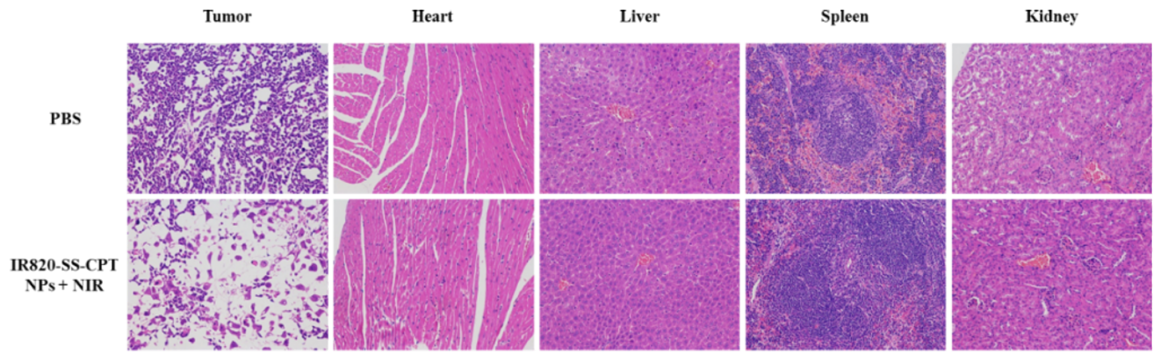


**Figure S20.** Representative H&E staining of the major organs and tumors of the mice treated with PBS and IR820-SS-CPT NPs + NIR.
